# Chromatin Remodeling Subunit ARID1A Negatively Regulates the Malignant Progression of Gastrointestinal Stromal Tumors by Targeting the MEMO1 Promoter

**DOI:** 10.7150/ijms.136256

**Published:** 2026-08-11

**Authors:** Yuhao Wang, Yi Shi, Shu Wang, Guanghui Xu, Ahui Fan, Liu Yutong, Haoyuan Wang, Yan Zhao, Chaosheng Peng, Yuxuan Ma, Yongzhan Nie, Jianjun Yang

**Affiliations:** 1 Department of Digestive Surgery, Xijing Hospital, Fourth Military Medical University, Xi'an, China.; 2 State Key Laboratory of Holistic Integrative Management of Gastrointestinal Cancers and Xijing Hospital of Digestive Diseases, Fourth Military Medical University, Xi'an, China.; 3 Department of Occupational and Environmental Health and the Ministry of Education Key Lab of Hazard Assessment and Control in Special Operational Environment, School of Public Health, Fourth Military Medical University, Xi'an, China.

**Keywords:** Gastrointestinal stromal tumors (GISTs), AT-rich interaction domain 1A (ARID1A), Mediator of cell motility 1 (MEMO1), Malignant progression, MAPK

## Abstract

Gastrointestinal stromal tumors (GISTs) are the most common sarcomas of the alimentary tract and are primarily characterized by malignant progression, a major cause of mortality. AT-rich interaction domain 1A (ARID1A), a core component of the chromatin-remodeling SWI/SNF complex, has been found to correlate with GIST tumor grade, although the underlying mechanism remains unclear. Its frequent inactivation across diverse cancer types reveals pleiotropic roles that intersect multiple hallmarks of cancer. In this study, we aimed to investigate the potential relationship between ARID1A and malignant progression in GISTs, as well as the underlying mechanism. Western blotting, real-time polymerase chain reaction, and immunohistochemistry were used to assess ARID1A expression in GIST tissues. Cell Counting Kit-8 (CCK-8) assays were performed to evaluate cell proliferation. Wound-healing and Transwell assays were conducted to assess cell migration and invasion. Flow cytometry was used to analyze apoptosis and cell cycle distribution. Label-free quantitative proteomics and chromatin immunoprecipitation sequencing (ChIP-seq) were employed to identify top candidate downstream targets of ARID1A. ARID1A expression was decreased in high-risk GIST tissues. Furthermore, ARID1A knockdown in GIST cells promoted proliferation and metastasis both *in vitro* and *in vivo*, and led to reduced apoptosis and impaired cell cycle arrest. We further demonstrated that ARID1A suppresses GIST proliferation and metastasis by inhibiting MEMO1 expression and inactivating the ERK1/2 signaling pathway. Notably, this regulatory axis was observed in KIT-null GIST cells, indicating that the ARID1A-MEMO1 pathway may function independently of canonical KIT signaling. Thus, ARID1A inhibits malignant progression in GISTs, providing new insights into its role in the prevention and treatment of human GISTs and suggesting its potential as a biomarker of malignant progression in GISTs.

## 1. Introduction

GISTs are the most common sarcomas of the gastrointestinal tract^1^-^3^. Although considered rare, micro-GISTs are quite common in autopsies, leading to the frequent underestimation of GISTs incidence^4^, ^5^. Prognosis depends on tumor size, mitotic count, and anatomic location according to the NIH risk stratification system. In advanced or metastatic disease, malignant progression and acquired multidrug resistance remain major clinical challenges that limit the durability of current treatments 6, 7. Genetic alterations are well-established drivers of cancer development and progression^8^-^10^. In GISTs, complex molecular heterogeneity leads to marked variability in tumor progression risk, complicating treatment selection^11^-^13^. Therefore, improving GIST management requires deeper insight into their regulatory networks, as the molecular mechanisms underlying tumorigenesis and malignant progression remain incompletely understood.

ARID1A is a tumor suppressor that participates in chromatin remodeling and serves as a core subunit of the switch/sucrose non-fermentable (SWI/SNF) complex^14^, ^15^. The SWI/SNF complex facilitates DNA accessibility for nuclear proteins and their regulatory complexes^16^, ^17^. Mutations in SWI/SNF complex components have been identified across multiple cancer types^18^-^20^. In metrial carcinomas, mutations in ARID1A in cells with phosphatase and tensin homolog deficiency lead to inactivation of the ARID1A/transforming growth factor β axis and promote invasion and migration 21. ARID1A function varies by hepatocellular carcinoma (HCC) stage 22. In HCC mouse models, sustained ARID1A expression promotes primary tumor formation, whereas its loss impairs later tumor progression. This apparent paradox reflects ARID1A's context-dependent function—its role varies by tissue type, genetic background, and disease stage 23. In HCC, ARID1A supports early tumorigenesis under specific genetic conditions; by contrast, ARID1A loss drives aggressive progression and poor prognosis in colorectal, gastric, and cholangiocarcinoma^24^-^26^. Thus, ARID1A's functional impact is highly context-specific—and its role in GISTs had remained unexplored until now.

Mediator of ERBB2-driven cell motility 1 (MEMO1) is a highly conserved 287-amino acid protein initially identified as a key mediator of ERBB2-induced cell migration. It has since been shown to promote epithelial-mesenchymal transition (EMT), enhance cell motility, and facilitate invasion across multiple cancer types 27. Elevated MEMO1 expression has been observed in breast and colorectal cancers, where it correlates with poor prognosis and aggressive tumor behavior 28. Moreover, in colorectal cancer, a transcription factor complex comprising the aryl hydrocarbon receptor (AhR) and AhR nuclear translocator (ARNT) directly binds the MEMO1 promoter to drive its expression in response to HER2 signaling 29. However, whether MEMO1 is regulated by other transcription factors remains unclear, and its role in GISTs has not been investigated.

Herein, we investigate ARID1A expression in GIST tissues and elucidate its role in regulating malignant progression. We demonstrate that ARID1A is decreased in high-risk GISTs and that—by downregulating MEMO1 expression and inactivating the ERK1/2 signaling pathway—it suppresses GIST proliferation and metastasis. Notably, we observed this regulatory axis in KIT-independent GIST cells, suggesting a potential KIT-bypass route. Our findings indicate that ARID1A may serve as a prognostic biomarker in GISTs and that ERK1/2 inhibitors could represent an effective therapeutic strategy for GIST patients.

## 2. Materials and methods

### 2.1. Cell lines and reagents

The GIST-T1 and GIST-48B cell lines were obtained from the University of the Chinese Academy of Sciences. GIST-T1 was established from a metastatic human GIST harboring a heterozygous 57-base deletion in KIT exon 11. GIST-48B harbors primary and secondary KIT mutations—V560D in exon 11 and D820V in the activation loop of exon 17—and exhibits undetectable KIT protein expression. GIST cells were maintained in IMDM (Gibco) supplemented with 10% fetal bovine serum (FBS; Gibco, New Zealand), 1% L-glutamine (Gibco), 1% antibiotic-antimycotic solution (Gibco), and 1% non-essential amino acids (Gibco) under controlled conditions of 5% CO₂ at 37 °C.

### 2.2. RNA interference

Specific ARID1A siRNAs were synthesized by Tsingke (Beijing, China). Transfection into cells was performed using Lipofectamine RNAiMAX (Invitrogen). After 24 h, siRNA-mediated knockdown efficiency was assessed by qRT-PCR and Western blotting.

### 2.3. Lentivirus infection

Sh-ARID1A lentivirus was obtained from GeneChem (Shanghai, China). Target cells (4 × 10⁶) were infected with 5 × 10⁷ transducing units (TU). An empty lentiviral vector served as the negative control.

### 2.4. Immunofluorescence staining

Cells were plated onto glass coverslips and fixed with 4% paraformaldehyde. Rabbit anti-human ARID1A primary antibody (Abcam, ab272905, 1:100) was applied at 4 °C overnight. Goat anti-rabbit secondary antibody was incubated at room temperature for 2 h.

### 2.5. RNA extraction and quantitative reverse transcriptase-polymerase chain reaction (qRT-PCR)

Total RNA was isolated using the GeneJET RNA Purification Kit (Thermo Scientific, USA). Gene expression levels were quantified using TB Green® Premix Ex Taq™ II (Takara) on a StepOnePlus™ Real-Time PCR System. Data were analyzed using the 2^(-ΔΔCt) method. ACTB (β-actin) was used as the endogenous reference gene for normalization. All qPCR reactions were performed with an annealing temperature of 60 °C, and amplicon lengths are listed in Supplementary Table 2.

### 2.6. Western blotting

Total protein was extracted using RIPA buffer (Beyotime Biotechnology). A total of 50 μg of protein was separated by SDS-PAGE (Epizyme) and subsequently transferred to NC membranes (PALL). The blots were incubated with secondary antibodies (Cell Signaling Technology). Detection of the bands was performed using ECL solution (UltraSignal). GAPDH or Tubulin was used as the control. The primary antibodies used were as follows: GAPDH (~36 kDa, rabbit monoclonal, Abclonal #A19056, 1:1000 for WB), ARID1A (~250 kDa, rabbit monoclonal, Abcam #ab272905, 1:400 for WB, 1:200 for IHC), MEMO1 (~30 kDa, rabbit polyclonal, Abclonal #A8582, 1:400 for WB), Tubulin (~55 kDa, rabbit monoclonal, Abclonal #A12289, 1:1000 for WB), ERK (~42 kDa, rabbit monoclonal, Abclonal #A4782, 1:400 for WB), and p-ERK (~44 kDa, rabbit monoclonal, Abclonal #A9451, 1:200 for WB). The secondary antibodies used were as follows: HRP-conjugated goat anti-rabbit IgG (Proteintech, #SA00001-2, lot: 20000428) and HRP-conjugated goat anti-mouse IgG (Proteintech, #SA00001-1, lot: 20000376), both used at 1:5000 dilution for Western blotting.

### 2.7. ChIP-seq Analysis

GIST-48B cell lines were crosslinked at room temperature with 1% formaldehyde for 10 minutes and then quenched by adding 125 mM glycine. The genomic DNA was subsequently digested into fragments ranging from 100 to 500 bp using micrococcal nuclease (M0247S, NEB) in a digestion buffer. Following this, 5 μg of an anti-ARID1A antibody was added to the sheared chromatin, and the mixture was rotated overnight at 4 °C. The samples were then incubated with protein A/G beads at 4°C for an additional 4-6 hours. After adding proteinase K, the protein-DNA complexes were decrosslinked. The resulting de-crosslinked DNA was isolated using a MinElute PCR Purification Kit (28004, Qiagen) and analyzed via ChIP-seq or ChIP-qPCR methods. Details regarding the primer sequences used in ChIP-qPCR and qPCR reactions can be found in Supplementary Table 2. Library preparation was performed using the KAPA Hyper Prep Kit (KK8502, KAPA Biosystems), and library sequencing was carried out by Saicheng Biotech (Guangzhou, China).

### 2.8. Label-Free Quantitation Mass Spectrometry and data analysis

Following SDS-PAGE separation, the gel region corresponding to the protein of interest was excised and cut into approximately 1 mm³ pieces for in-gel trypsin digestion and subsequent mass spectrometry analysis. Mass spectrometry data were acquired using the Q Exactive HF mass spectrometer (Thermo Fisher Scientific, Bremen, Germany) coupled to an UltiMate 3000 RSLCnano liquid chromatography system (Thermo Fisher Scientific, Sunnyvale, CA, USA).

### 2.9. Immunohistochemistry (IHC)

Sections were dewaxed and rehydrated. After blocking for 10 minutes, the sections were incubated, followed by blocking and incubation with primary antibodies, including anti-ARID1A. Subsequently, the slides were treated with HRP secondary antibody. Each section was also stained with hematoxylin and eosin on initial slides to confirm the diagnosis. The results were evaluated using the H-score system. The staining intensity was scored from 0 to 3 (0 = no staining; 1 = weak; 2 = moderate; 3 = strong). The percentage of positive tumor cells was scored as 0 (< 5%), 1 (5-25%), 2 (26-50%), 3 (51-75%), or 4 (> 75%). The final H-score for each slide was calculated as the sum of (intensity score × percentage score) across all staining intensities. The final score of each slide was calculated as the average score of the 5 fields selected randomly and ranged from 0 to 12 (intensity score x percentage score). The cutoff value (H-score = 8) for defining low versus high ARID1A expression was determined using X-tile software (Yale University, New Haven, CT, USA, version 3.6.1), a tool specifically designed for survival-based biomarker cutoff optimization. Briefly, the H-scores from the TMA cohort (n = 69) and corresponding overall survival data were entered into the software. X-tile then systematically evaluated each possible H-score value as a potential cutoff and identified the value that maximized the statistical significance of the difference in survival between the two groups (i.e., the lowest log-rank test P value). This method ensures an objective, data-driven determination of the cutoff value 30. Specifically, low expression of ARID1A was defined as a final score less than 8 (IHC score < 8, 50th percentile of ARID1A IHC score, according to bioinformatic analysis).

### 2.10. Tissue microarrays (TMA)

Tissue microarray construction was performed using samples obtained from Shanghai Outdo Biotech Co., Ltd. (cat. no. HDgS-GISTs090Loc-01). Subsequently, 4 μm sections were cut and stained with hematoxylin and eosin on the initial slides to confirm the diagnosis. The slides were then used for immunohistochemistry assays. The TMA comprises GIST cases diagnosed from 2010 to 2024, with complete clinical follow-up and risk grading per the modified NIH consensus criteria. TMA cores (two per case) were sampled from representative tumor areas. IHC scoring was performed independently by two pathologists blinded to clinical data.

### 2.11. *In vivo* tumorigenicity

GIST-T1 cells (1 × 10^7^ cells in 0.15 mL of PBS) transfected with GV493 (pFU-GW-016) vector or empty vector were injected subcutaneously into the dorsal flank of 6-week-old female Balb/c nude mice. Mice were randomly allocated to the experimental (sh-ARID1A) or control (sh-NC) groups using a computer-generated random number sequence before cell injection. Tumor diameter was measured every 5 days over a period of 30 days. All animal study protocols were approved by the Institutional Animal Care and Use Committee of the Fourth Military Medical University (Approval No. 20230239), and all procedures were performed in accordance with the relevant guidelines and regulations.

### 2.12. CCK8 assay

The same batch of 48B and T1 ARID1A knockdown cells, along with control cells in the logarithmic growth phase, was seeded into a 96-well plate. Subsequently, the CCK-8 assay (InCellGene, TX, USA) was employed to assess the proliferation capabilities of the stable cell lines at the time points of 0 h, 24 h, 48 h, 72 h, and 96 h. Each well received 10% CCK-8 solution and was incubated for 1 hour at 37 °C. The CCK-8 assay measures cell viability based on the reduction of WST-8—a water-soluble tetrazolium salt—into an orange-yellow formazan dye by dehydrogenases in viable cells. Absorbance was measured at 450 nm using a microplate reader.

### 2.13. Wound healing assay

Cells were plated into six-well plates and allowed to grow until confluence was reached. Two separate parallel wounds were then created by scratching the cell layer with a 200 μL plastic pipette tip. The migration distance of 48B cells was observed and imaged under an Olympus CX22 microscope (Shanghai) at time points of 0 h and 72 h, while T1 cells were assessed at 0 h and 48 h. These experiments were conducted in triplicate.

### 2.14. Transwell assay

For migration and invasion assays, GIST cells (1 × 10^6^ cells in 200 µL of serum-free IMDM) were seeded into the upper Transwell chamber. The lower chamber contained 600 µL of IMDM supplemented with 10% FBS as a chemoattractant. For invasion assays, the upper chamber inserts were pre-coated with Matrigel (Corning) at 37 °C for 4 hours before cell seeding. After 24 hours of incubation, non-migrating or non-invading cells on the upper surface were removed with a cotton swab, and cells on the lower membrane surface were fixed, stained with 0.1% crystal violet, and counted under a light microscope.

### 2.15. Cell cycle analyses

Cells were seeded into 6-well plates at a specified density per well. After 24 hours of culture, cell cycle analysis was conducted using flow cytometry with a detection kit (Elabscience, China) and the FACSCalibur BD flow cytometer.

### 2.16. Cell apoptosis analysis

After centrifugation, the supernatant was discarded, and the cells were resuspended in 500 µL of 1× Annexin V binding buffer. Subsequently, 5 µL of Annexin V-PE and 5 µL of 7-Amino Actinomycin D (Elabscience, China) were added. The apoptosis rate was analyzed and calculated using flow cytometry.

### 2.17. Ethics Statement

The TMA (cat. no. HDgS-GISTs090Loc-01) was obtained from Shanghai Outdo Biotech Co., Ltd. and approved by the Ethics Committee of Fourth Military Medical University (No. KY20213525-1). All patients provided written informed consent, and TMA use complied with the Declaration of Helsinki. We also included 20 fresh-frozen GIST tissues (10 high-risk, 10 low-risk) collected at our institution (Fourth Military Medical University), approved by its IRB (No. KY20213525-1), with patient consent. All animal experiments were performed under protocols reviewed and approved by the Institutional Animal Care and Use Committee of the Fourth Military Medical University (Approval No. 20230239), and all procedures were carried out in accordance with the relevant guidelines and regulations.

### 2.18. Statistical analysis

All data are presented as the mean ± standard deviation from at least three independent experiments. Differences between two groups or among three or more groups were analyzed using Student's t-test or one-way ANOVA, respectively. The relationship between ARID1A expression levels and clinical parameters was assessed using the chi-square test.

## Results

### 3.1. ARID1A downregulation is associated with high-risk grade and poorer survival outcomes in GISTs

**Figure 1:**
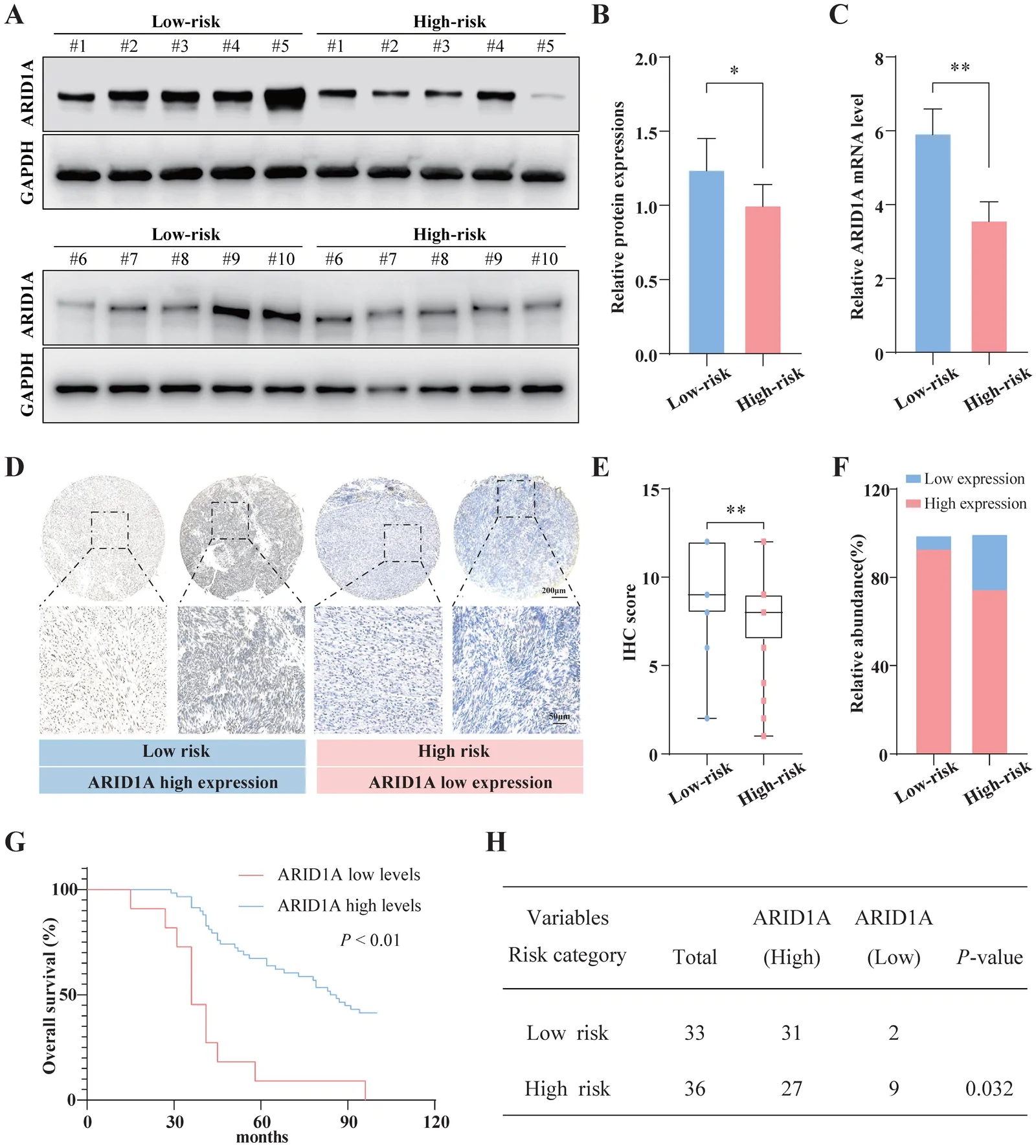
** ARID1A expression is negatively correlated with the malignant progression of GISTs. A.** ARID1A expression in low-risk and high-risk GIST tissues was determined by Western blotting (n = 10). **B.** Relative ARID1A protein levels in GIST tissues were detected by Western blotting (n = 10). **C.** Relative ARID1A mRNA levels in GIST tissues were detected by real-time polymerase chain reaction (n = 10). **D.** ARID1A expression was measured in TMA that contains 69 samples of GISTs using IHC. **E.** ARID1A expression H-scores were assessed by IHC in a TMA containing 69 GIST samples. **F**. The relative abundance of ARID1A expression was analyzed in GISTs of high-risk and low-risk grade. **G.** Survival analyses in GIST patients cohort according to ARID1A expression (n = 69). **H.** The association of expression levels of ARID1A with the clinical features of GISTs was calculated using Pearson's χ^2^ test or Fisher exact test.

To determine the protein and mRNA levels of ARID1A in GISTs with different risk grades, we investigated ARID1A expression in 10 GIST tissues of high-risk and 10 low-risk GIST tissues. The data showed that ARID1A expression was significantly lower in high-risk GIST tissues (Fig. 1A, 1B). Consistent with ARID1A mRNA levels, ARID1A protein levels were decreased in GIST samples of high-grade compared with their low-risk counterparts (Fig. 1C). A tissue microarray (TMA) was used to confirm the expression profile and the result showed that ARID1A, primarily located in the nucleus, was remarkably downregulated in high-risk GIST tissues (Fig. 1D, 1E). Additionally, 93.93% of patients in the low-risk group had high ARID1A expression, while 6.06% had low ARID1A expression. In the high-risk group, 75.00% of patients had high ARID1A expression, and 25.00% had low ARID1A expression (Fig. 1F). Kaplan-Meier survival curves indicate that patients with low ARID1A expression are associated with significantly worse prognosis (Fig. 1G, log-rank test, *P* < 0.01). IHC analysis further revealed that ARID1A loss was strongly correlated with risk grade, tumor size and location (Fig. 1H and Table 1, Risk category,* P* = 0.032; Tumor size, *P* = 0.020; Tumor location, *P* = 0.040). Low ARID1A expression remained an independent and statistically significant predictor of worse overall survival (Table S1, HR = 0.382, 95% CI: 0.14-0.81, *P* = 0.010). Taken together, ARID1A expression was significantly reduced in high-risk GIST tissues, and its downregulation was independently associated with poorer survival outcomes in patients with GISTs.

### 3.2. Decreased ARID1A in GIST cells promotes proliferation, migration and invasion

**Figure 2:**
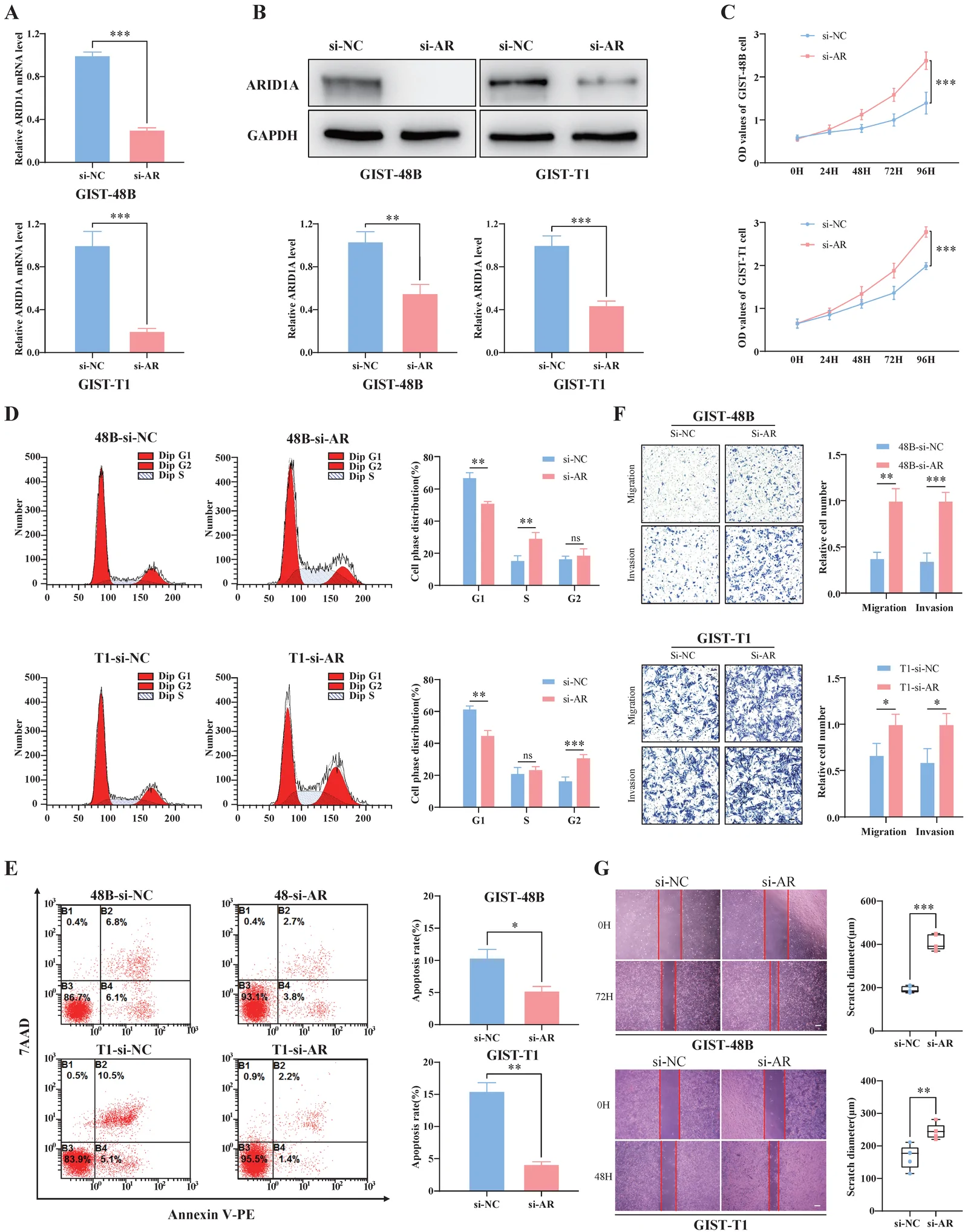
** Silencing of ARID1A promotes the proliferation, migration and invasion of GIST cells. A.** ARID1A mRNA levels were examined using qRT-PCR in GIST cells (n=3).** B.** ARID1A protein levels were examined using Western blotting (n=3). **C.** The viability of GIST cells was detected by CCK8 assay (n=5). **D.** The cell cycle distribution of GIST cells was detected using flow cytometry analysis (n=3). **E.** Apoptosis in GIST cells was analyzed using flow cytometry (n=3). **F.** Cell migration and invasion were assessed by Transwell insert (bar=50 μm, n=3). **G.** The ability of migration and proliferation in GIST cells was tested with scratch test (bar=200 μm, n=5). si-NC, negative control group. si-AR, GIST cells transfected with si-ARID1A. The data in the bar plots are expressed as the mean ± SD. * P < 0.05, ** P < 0.01, *** P < 0.001. ns, not significant.

To investigate the biological function of ARID1A, we silenced ARID1A in GIST-T1 and GIST-48B cells with small interfering RNA. The transfection efficiency was confirmed by real-time PCR and western blotting (Fig. 2A, 2B). The CCK8 assays revealed that cell proliferation was significantly promoted after ARID1A silencing in T1 and 48B cells (Fig. 2C). Moreover, knockdown of ARID1A in GIST cells drove the cell cycle into S phase and G2 phase and reduced the population of cells in G1 phase compared with the control (Fig. 2D). Moreover, we performed flow cytometry to determine whether ARID1A regulates GIST cell apoptosis and the cell cycle. Knockdown of ARID1A was associated with a significant reduction in apoptotic cells, as assessed by flow cytometry (Fig. 2E). Additionally, Transwell and wound-healing assays indicated that the loss of ARID1A promoted cell migration and invasion *in vitro*. (Fig. 2F, 2G). These results demonstrated that ARID1A could inhibit the proliferation, migration, and invasion of GIST cells.

### 3.3. ARID1A silencing promotes GIST cells growth *in vivo*

**Figure 3:**
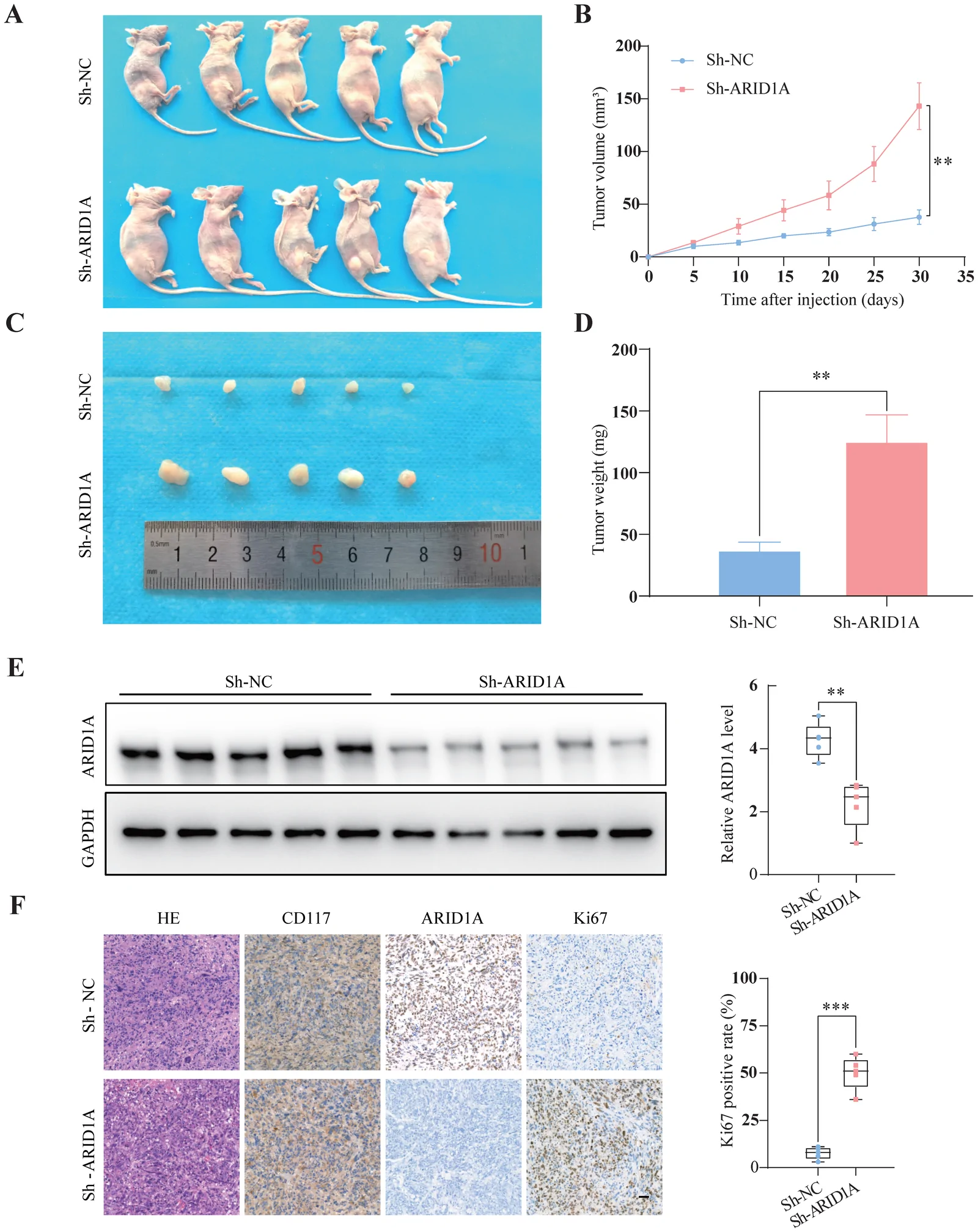
** ARID1A depletion promotes GISTs xenograft tumor growth *in vivo*. A.** GIST-T1 cells stably expressing sh-ARID1A or scrambled control shRNA were subcutaneously injected into nude mice (n=5). ARID1A-silenced GIST-T1 cells formed larger tumors compared with cells expressing the scrambled control after 30 days. **B.** Tumor growth curves (n=5). **C.** Representative images of xenograft tumors (n=5). **D.** Tumor weight (n=5). **E.** ARID1A expression in xenograft tumors was determined by Western blot (n=5). **F.** HE and immunohistochemical staining for CD117, ARID1A, and Ki67 in xenograft tumors (bar=50 μm, n=5). T1-NC, negative control group. Sh-ARID1A, GIST cells transfected with sh-ARID1A. The data in the bar plots are expressed as the mean ± SD. ** *P* < 0.01, *** P < 0.001.

To investigate the impact of ARID1A on the tumorigenic behavior of GISTs *in vivo*, we performed xenograft experiments in nude mice. Specifically, GIST-T1 cells stably expressing shARID1A or scrambled control shRNA were subcutaneously injected into the dorsal flanks of mice. Depletion of ARID1A resulted in a significant enhancement of tumor growth (Fig. 3A). Tumors derived from ARID1A-knockdown cells exhibited significantly accelerated growth rates (Fig. 3B) and were notably heavier in weight compared to control tumors (Fig. 3C, 3D). Additionally, western blotting and IHC analyses confirmed reduced ARID1A expression levels in ARID1A-knockdown tumors relative to control tumors (Fig. 3E, 3F). Moreover, we observed an increased Ki67 (proliferation marker) positivity rate in tumor tissues from the ARID1A-knockdown group compared to the control group (Fig. 3F). Collectively, these findings suggest that ARID1A exerts an inhibitory effect on GISTs tumor growth *in vivo*, consistent with its role as a tumor suppressor in GISTs progression.

### 3.4. Genome-wide annotation of ARID1A binding sites in GIST cells

**Figure 4:**
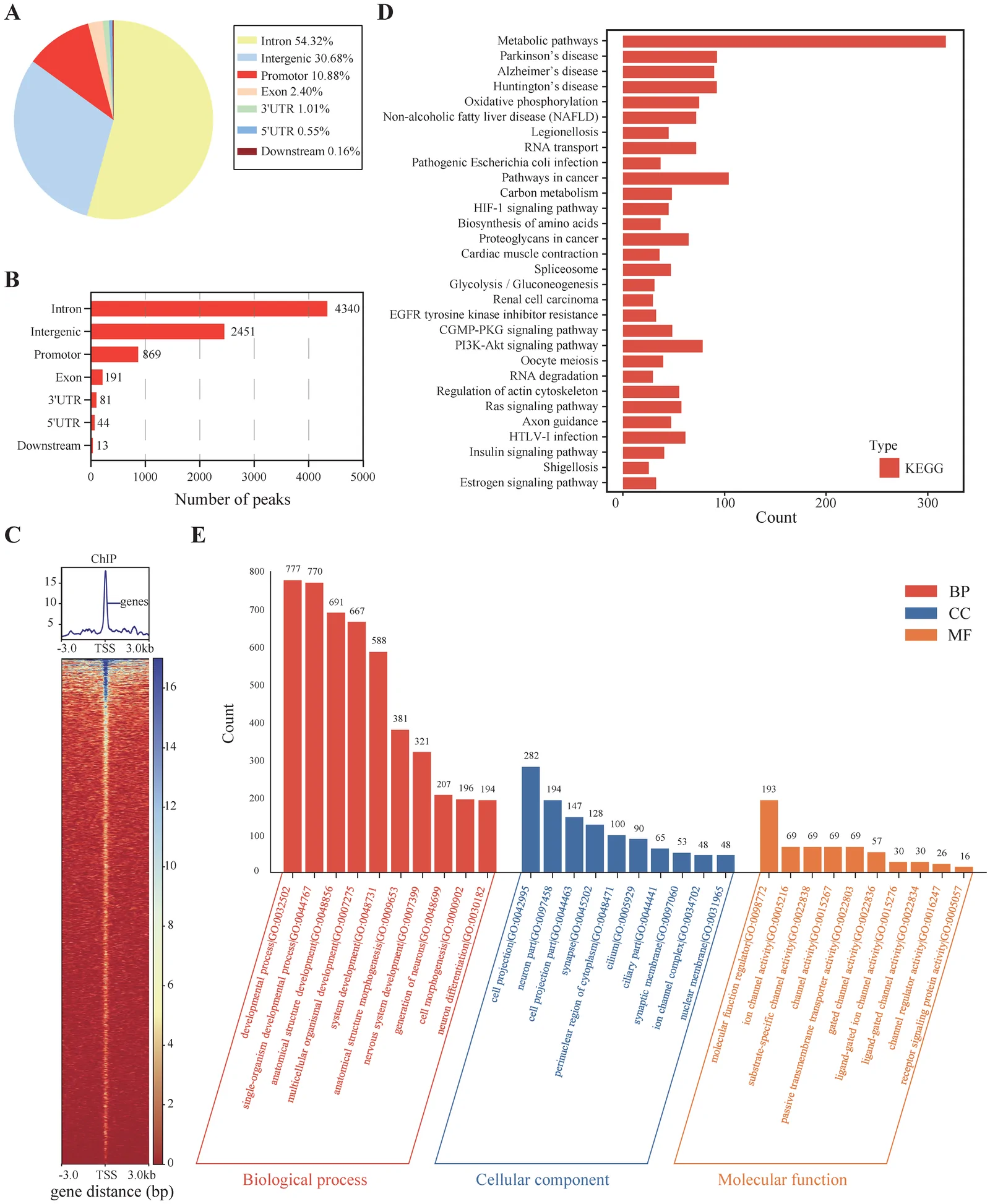
** Genome-wide annotation of ARID1A binding sites in GIST cell. A.** The distribution of ARID1A peaks among the genome was shown using pie chart. **B.** The number of ARID1A peaks among the genome was shown using bar chart. **C.** The co-binding character of ARID1A was shown in the form of distributing patterns of the overlapping regions. **D.** KEGG pathway enrichment from the ARID1A ChIP-Seq samples. **E.** GO enrichments from the ARID1A ChIP-Seq samples.

To elucidate the potential targets of ARID1A, genomic DNA fragments associated with ARID1A were isolated using a specific anti-ARID1A antibody, followed by chromatin immunoprecipitation to characterize these fragments. Analysis of ChIP-seq data revealed 54.2 million short reads obtained from ARID1A-immunoprecipitated samples. We identified 7,989 unique regions within the ARID1A ChIP-seq dataset (Supplementary Table 3). Bowtie software was employed to align these reads against the human reference genome. ChIP-seq signals demonstrated significant enrichment at peak centers across samples compared to input controls (Figure S1). To annotate the target genes, ChIPSeeker and MACS2 were used to annotate the peak regions, which spanned from 3 kb upstream to 3 kb downstream of the transcription start site (TSS). The annotated genes showed a distribution pattern where 7,989 binding sites were located within the range of the TSS. Specifically, 4,340 sites (54.32%) were in introns, 2,451 sites (30.68%) were intergenic, 869 sites (10.88%) were in promoters, 191 sites (2.40%) were in exons, 81 sites (1.01%) were in 3' UTRs, and 44 sites (0.55%) were in 5' UTRs. The remaining 13 binding sites (0.16%) were located downstream of the TSS (Fig. 4A-C). These results indicated that ARID1A predominantly binds to introns, followed by intergenic regions, promoters, exons, 3' UTRs, and 5' UTRs. In addition to these binding sites, a small proportion of binding sites were located downstream of the TSS (Fig. 4B). KEGG analysis showed that these genes were involved in metabolic pathways, neuron-related diseases, tyrosine kinase-related pathways, pathways in cancer, and signaling pathways that regulate the biological behavior of malignancy, such as the PI3K-AKT signaling pathway, among others (Fig. 4D). GO analysis was performed on the genes recognized by ARID1A, and these genes were classified into various functional categories. The top categories included biological processes, developmental processes, single-organism development, and others (Fig. 4E). Collectively, these findings suggest that ARID1A functions as a crucial epigenetic regulator, potentially driving the malignant progression of GISTs by modulating key oncogenic signaling cascades and developmental processes.

### 3.5. ARID1A negatively regulates MEMO1 by interacting with its promoter region

**Figure 5:**
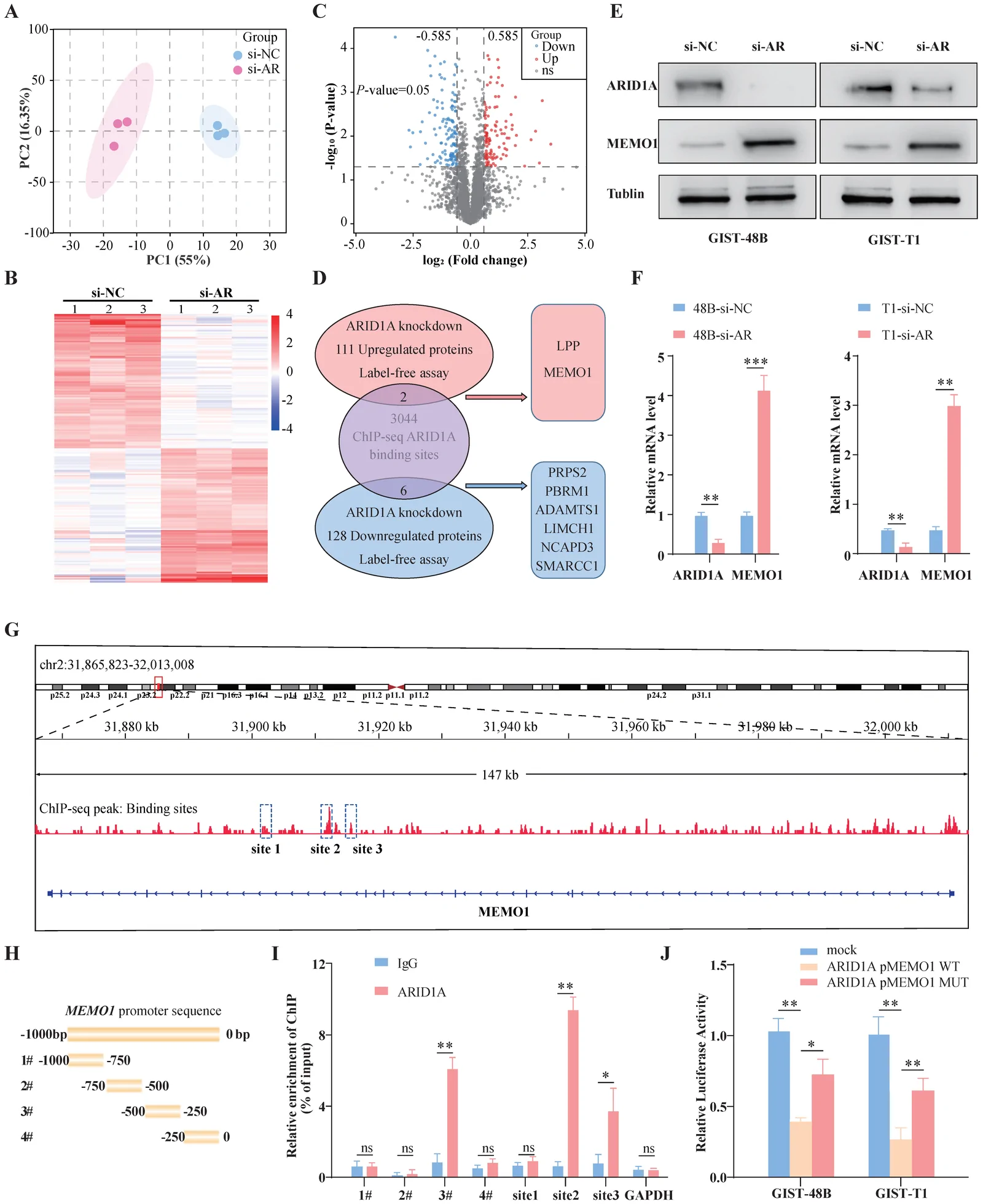
** ARID1A negatively regulates MEMO1 expression in GIST cells. A.** The PCA score of si-NC and si-AR groups showed a significant difference. **B.** The differentially expressed proteins of si-NC and si-AR groups were shown using heat map (top 100). **C.** The differentially expressed proteins of si-NC and si-AR groups was shown using volcano map. **D.** Intersection genes from ARID1A targeted ChIP-seq data and ARID1A knockdown label-free data. **E.** ARID1A protein levels were examined using Western blotting (n=3). **F.** ARID1A mRNA levels were examined using qRT-PCR (n=3). **G.** Binding peaks of MEMO1 and ARID1A were detected in three sites of MEMO1 sequence. **H.** Four primers were designed based on MEMO1 promoter sequence. **I.** Enrichment of MEMO1 promoter fragments using an antibody against ARID1A was assessed by ChIP-qPCR analysis. **J.** Dual luciferase reporter assays were performed with GIST cells co-transfected with pMEMO1 wild-type (WT) or mutated pMEMO1 and with ARID1A overexpression or an empty vector. The data in the bar plots are expressed as the mean ± SD. * *P* < 0.05, ** *P* < 0.01, *** P < 0.001. ns, not significant.

To investigate the genes that may be regulated by ARID1A, Label-free proteomics analysis was performed on GIST-48B cells transfected with siRNA for ARID1A knockdown. Principal component analysis (PCA) revealed that the overall protein expression profile of the si-ARID1A group was clearly separated from the si-NC group (Fig. 5A). Based on the label-free analysis between these two groups, 111 upregulated proteins and 128 down-regulated proteins were identified (Supplementary Tables 4 and 5, Fig. 5B, 5C). To confirm whether these differentially expressed proteins are regulated by ARID1A, the ChIP-seq and Label-free data were analyzed both in GIST-48B cells. Among the 239 differentially expressed proteins, ARID1A was found to bind to the gene sequences of 8 of them (Fig. 5D). Subsequently, qRT-PCR and Western blotting were conducted to verify that MEMO1 could be regulated by ARID1A and the results showed that ARID1A negatively regulates MEMO1 (Fig. 5E, 5F). The ChIP-seq data and the JASPAR database were used to identify three potential ARID1A binding sites in the MEMO1 sequence (Fig. 5G). We verified the ChIP-seq results using quantitative PCR (ChIP-qPCR). Primers were designed based on the candidate binding sites and the MEMO1 promoter sequence (Supplementary Table 2, Fig. 5G). Using the predicted MEMO1 promoter sequence that was expected to interact with ARID1A, primers were designed (Fig. 5H). The ChIP-qPCR assay showed that MEMO1 was more highly enriched compared to the control IgG, indicating that the ARID1A protein binds to the potential binding sites in the MEMO1 promoter (Fig. 5I). The wild-type MEMO1 promoter region—containing the putative ARID1A-binding site identified by ChIP-PCR—and a corresponding mutated version, with targeted mutations in the binding motif, were cloned into a luciferase reporter vector. Following co-transfection of ARID1A into GIST cells, we observed a marked decrease in luciferase activity under the control of the wild-type promoter. This transcription inhibition was entirely eliminated when the binding site was mutated (Fig. 5J). Taken together, these findings indicated that ARID1A negatively regulates MEMO1 by interacting with its promoter region.

### 3.6. MEMO1 knockdown reverse the malignant progression induced by ARID1A loss

**Figure 6:**
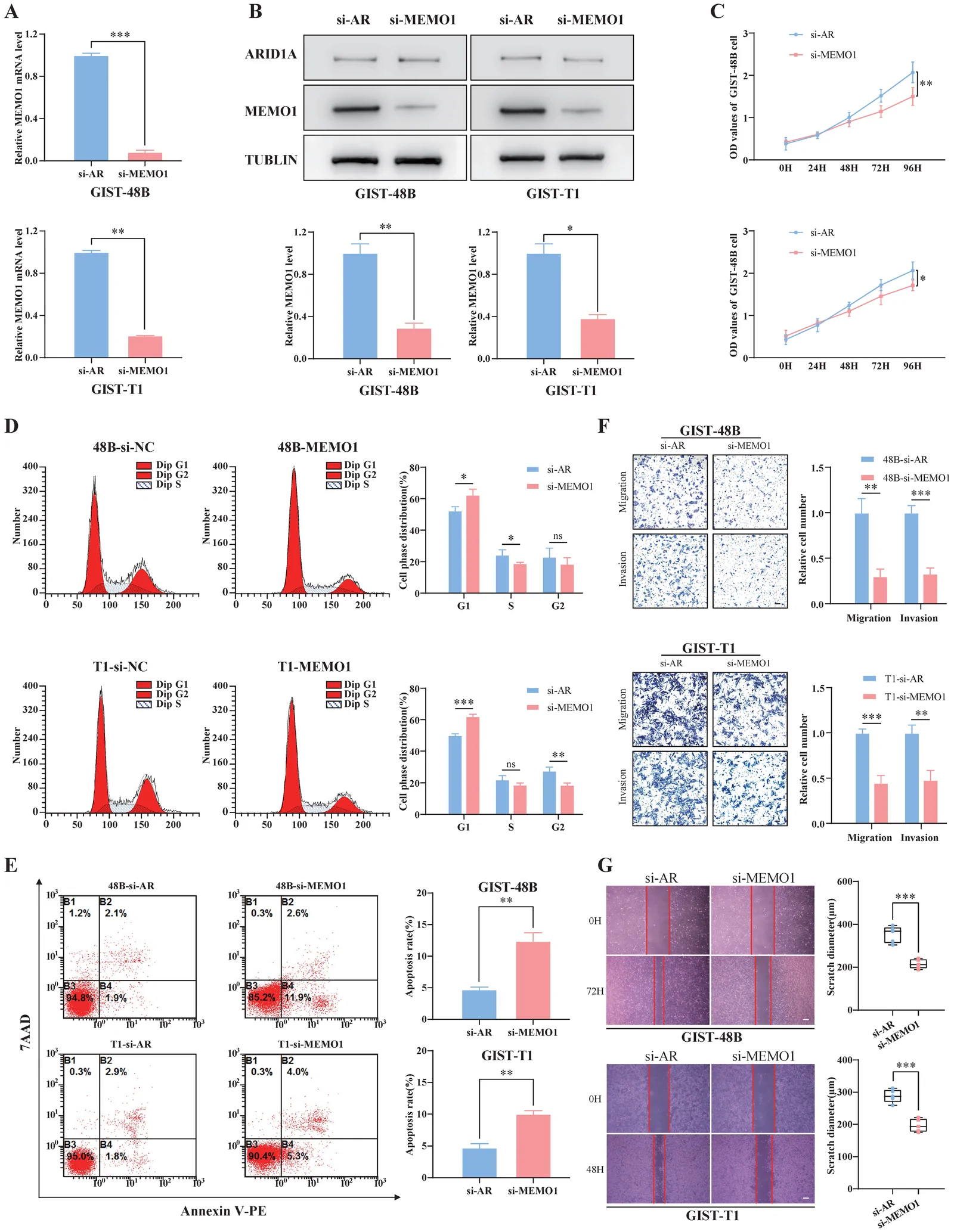
** MEMO1 knockdown can inhibit the proliferation, migration, and invasion of GIST cells. A.** MEMO1 mRNA levels were examined using qRT-PCR in GIST cells (n=3). **B.** MEMO1 protein levels were examined using Western blotting (n=3). **C.** The viability of GIST cells was detected by CCK8 assay (n=5). **D.** The cell cycle distribution of GIST cells was detected using flow cytometry analysis (n=3). **E.** Apoptosis in GIST cells was analyzed using flow cytometry (n=3). **F.** Cell migration and invasion were assessed by Transwell insert (bar=50 μm, n=3). **G.** The ability of migration and proliferation in GIST cells was tested with scratch test (bar=200 μm, n=5). si-AR, GIST cells transfected with si-ARID1A. si-MEMO1: GIST cells transfected both with si-ARID1A and si-MEMO1. The data in the bar plots are expressed as the mean ± SD. * *P* < 0.05, ** *P* < 0.01, *** P < 0.001. ns, not significant.

To validate the role of MEMO1, we conducted siRNA in GIST cells after ARID1A knockdown. Western blotting and qRT-PCR were used to confirm that MEMO1 expression was significantly decreased (Fig. 6A, 6B). The proliferation ability of GIST cells diminished after MEMO1 knockdown, as detected by the CCK-8 assay (Fig. 6C). Furthermore, flow cytometry data showed an increase in the apoptosis rate, and a decrease in cells in the S and G2 phases, indicating that cells were arrested in the G1 phase after MEMO1 knockdown (Fig. 6D, 6E). The Transwell assay also showed limited metastasis and invasion after si-MEMO1 transfection (Fig. 6F). To further validate the effect of MEMO1, a scratch assay was performed, and the results indicated that cells exhibited reduced metastatic ability after si-MEMO1 transfection (Fig. 6G). The results indicated that MEMO1 knockdown reverses the malignant progression induced by ARID1A knockdown.

### 3.7. ARID1A loss activates GIST cell proliferation, metastasis and invasion by stimulating MEMO1 through the MAPK pathway

**Figure 7:**
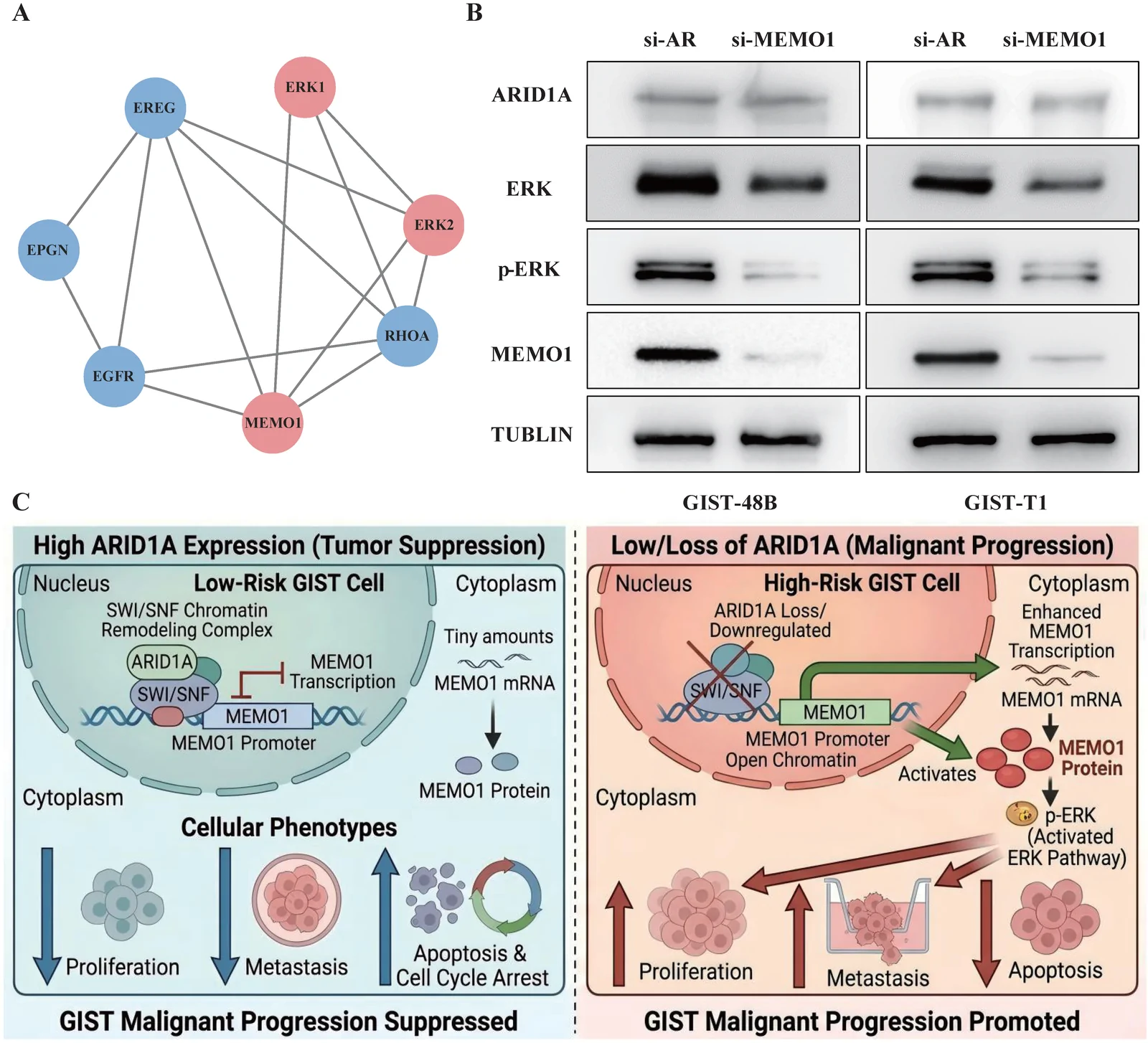
** ARID1A knockdown promotes the malignant progression of GISTs by regulating the MAPK signaling pathway through downstream MEMO1. A.** STRING was employed to predict effector downstream MEMO1. **B.** ERK1/2 and phosphorylated ERK1/2 were detected using Western blotting. **C.** The molecular mechanism of ARID1A-MEMO1-ERK1/2 axis and its effect in GISTs was visualized in graphical abstract.

Next, we verified the signaling pathway downstream of MEMO1. A protein-protein interaction network was constructed using the STRING database, and ERK1/2 and EGFR were identified as the top downstream candidates of MEMO1 (Fig. 7A). Subsequently, a decreased expression level of phosphorylated-ERK1/2 was observed following MEMO1 knockdown (Fig. 7B). The molecular mechanism of the ARID1A-MEMO1-ERK1/2 axis and its effect in GISTs was visualized. The MAPK pathway, which is downstream of KIT, is involved in the malignant progression of GISTs. ARID1A regulates this progression through the ARID1A-MEMO1-MAPK axis. ARID1A suppresses MEMO1 expression, thereby limiting the activation of the MAPK pathway. Taken together, these findings indicated that ARID1A knockdown leads to the upregulation of MEMO1, which in turn activates the MAPK signaling pathway, thereby promoting the malignant progression of GISTs (Fig. 7C).

## 4. Discussion

Our study suggests that ARID1A is an important regulator that is markedly downregulated in high-risk GIST tissues, and that knockdown of ARID1A promotes the malignant progression of GIST cells both *in vitro* and *in vivo*. Mechanistically, the effect of ARID1A downregulation on malignant progression was associated with activation of the MEMO1 and MAPK signaling pathways.

GISTs currently are a tremendous health burden on communities worldwide and is thought to result from a combination of genetic alterations. Despite sharing common mutations, such as *KIT* and *PDGFRA*, the potential for malignant progression varies among GISTs^3^, ^31^. For instance, GISTs associated with a high risk of aggressive progression typically exhibit the same *KIT* or *PDGFRA* mutations as those detected in micro-GISTs with an extremely low risk of progression^32^-^34^. This variability suggests that additional molecules regulate the biological behavior of GISTs independently of *KIT* and *PDGFRA*. Understanding the mechanisms and functions of these potential additional molecules is crucial to develop more effective therapeutic strategies.

Among the potential additional molecules mentioned earlier, ARID1A has recently emerged as a key player in GIST progression. It has been found to be highly correlated with GIST grade and to be involved in the transformation from low-risk to high-risk GISTs, thereby serving as a potential indicator of GIST progression 14. ARID1A is a core component of the ATP-dependent chromatin remodeling complex SWI/SNF and is widely recognized as a tumor suppressor. Depletion or mutation of ARID1A has been frequently observed in various types of cancer, highlighting its critical role in tumorigenesis and progression^35^, ^36^. In endometrial carcinomas, ARID1A deficiency leads to the loss of TGF-β tumor suppressive function, and inactivation of the ARID1A/TGF-β axis promotes migration and invasion in PTEN-deleted endometrial tumor cells 21. Additionally, ARID1A loss in hepatocellular carcinoma results in decreased chromatin accessibility and reduced transcription of genes associated with cellular migration, invasion, and metastasis 23. ARID1A functions as a tumor suppressor by modulating gene transcription, either by directly regulating cancer-related gene expression or indirectly influencing histone-modifying enzymes that add or remove modifications at regulatory regions^37^, ^38^. These findings indicate that ARID1A exerts regulatory functions in tumorigenesis and malignant progression. However, the extent to which and the mechanisms by which ARID1A influences the malignant progression of GISTs remain largely elusive. In the present study, we investigated ARID1A expression in GIST tissues and found that ARID1A was significantly downregulated in high-risk GIST tissues. Subsequent loss-of-function analyses revealed that ARID1A knockdown promoted GIST cell proliferation, migration, and invasion both *in vitro* and *in vivo*. Collectively, our findings demonstrate that ARID1A functions as a tumor suppressor protein in GIST cells.

Sun et al. reported that ARID1A knockdown increased the phosphorylation levels of several oncogenic proteins, including EGFR, ErbB2, and RAF1, thereby activating the corresponding signaling pathways and promoting disease progression in lung adenocarcinoma 30. In addition, only chromosomal-instability type gastric cancer with ARID1A deletion in the TCGA cohort could benefit from fluorouracil-based adjuvant chemotherapy and ARID1A deletion showed a trend of better responsiveness to immunotherapy and suggested a superior overall survival rate after anti-PD-1 immunotherapy 39. However, Xu et al. reported that ARID1A mutations and copy number losses are differentially enriched in distinct molecular subtypes of gastric cancer, indicating that the mechanisms underlying ARID1A deficiency may vary across these subtypes and ARID1A-loss gastric cancer patients were characterized by a favorable prognosis 40. Overall, ARID1A plays an important role in different cancer types through various mechanisms. The inconsistencies in the evaluation of ARID1A suggest that its underlying mechanisms require further investigation.

Our observation that the ARID1A-MEMO1-MAPK axis remains active in KIT-independent GIST cells raises a clinically relevant question. In the context of GIST, acquired resistance to TKIs such as imatinib, sunitinib, and regorafenib is frequently driven by secondary KIT mutations. However, emerging evidence indicates that MAPK pathway reactivation can also occur through KIT-independent mechanisms. Notably, Gupta and colleagues demonstrated that combining the KIT/PDGFRA inhibitor ripretinib with MEK inhibitors synergistically induced apoptosis and prevented the outgrowth of resistant colonies in both imatinib-sensitive and imatinib-resistant GIST cell lines 41. This preclinical evidence provides a rationale for the hypothesis that dual blockade of KIT and MEK may offer a strategy to overcome resistance. Our findings add a potential mechanistic layer to this therapeutic rationale: in tumors with ARID1A loss, MEMO1 upregulation and subsequent ERK/MAPK hyperactivation may sustain malignant progression even when KIT is effectively inhibited. Whether ARID1A-low GISTs are particularly sensitive to MEK inhibitor-based regimens, and whether such sensitivity is independent of KIT mutation status, are questions that warrant further investigation. If validated, ARID1A expression level could serve as a biomarker to guide patient selection for combined TKI and MEK inhibition strategies—a hypothesis that we believe merits further exploration in preclinical models and, ultimately, in clinical settings.

However, the precise mechanisms by which ARID1A regulates the malignant progression of GISTs remain to be elucidated. In this study, we report that the loss of ARID1A is correlated with the classification of GISTs risk and plays a role in the transformation from low-risk to high-risk GISTs through the ARID1A-MEMO1-MAPK axis, facilitating the malignant progression of GISTs. MEMO1, a 287-amino acid protein, plays a crucial role in the epithelial-mesenchymal transition (EMT) process in human colorectal cancers 42. It has also been observed to enhance migration and invasion capabilities in these cancers, hinting at its potential function as an oncogenic gene that influences tumorigenesis^43^, ^44^. To verify the role of MEMO1, the gene was knocked down in cells that had previously undergone ARID1A knockdown. The results indicated that MEMO1 knockdown restored the functional activity of ARID1A, resulting in decreased proliferation, metastasis, and invasion abilities in GIST cells, along with the induction of cell-cycle arrest. Subsequently, to identify the terminal effector of the ARID1A-MEMO1 axis, a protein-protein interaction network was constructed using databases such as STRING and GeneCards. ERK and EGFR were identified as top candidates for further examination. Western blotting was employed to assess the levels of ERK and phosphorylated ERK (p-ERK) after MEMO1 knockdown. The results demonstrated that the level of p-ERK was significantly reduced following MEMO1 knockdown. The RAS-RAF-MEK-ERK signaling pathway, which is evolutionarily conserved, plays a crucial role in cell proliferation and differentiation^45^, ^46^. Mutations in either RAS or RAF can result in the abnormal activation of tyrosine kinase-related pathways, which contribute to carcinogenesis 47. As an effector within the RAS-RAF-MEK-ERK pathway, ERK has the ability to phosphorylate numerous substrates such as transcription factors and protein kinases. This modulation subsequently influences the biological behaviors exhibited by cancer cells 48. Some studies suggest that the combination of TKIs and MEK inhibitors further reduces the activation of signaling pathways downstream of KIT 49. However, the molecular mechanism of the MEK pathway in GISTs remains poorly understood 50. Our study revealed that although GISTs originate from interstitial cells and MEK has traditionally been considered vital only in epithelium-derived tumors, MEK plays a significant role in GISTs, independent of KIT signaling. Through a series of experiments, we found that inhibiting MEK in GISTs led to a reduction in cell proliferation and migration, suggesting a novel therapeutic target for this type of cancer that has often been overlooked.

In this study, we investigate ARID1A expression in GIST tissues, and elucidate the role of ARID1A in regulating malignant progression in GISTs. However, there are still some limitations in this study. First, while we focused on the role of ARID1A in this study, ARID1A is an evolutionarily conserved gene involved in numerous biological processes through diverse mechanisms. While our *in vivo* data demonstrate a correlation between ARID1A loss and tumor growth, future studies utilizing inducible genetic rescue models *in vivo* are required to establish a definitive causal relationship. We are currently optimizing these technically demanding approaches in GIST models. Therefore, future studies should explore the additional roles of ARID1A in GISTs, particularly its functions beyond DNA binding. Second, although we found that MEMO1 interacts with the RAS-MAPK pathway, the specific role of EGFR, which may be regulated by MEMO1, and the downstream effects of RAS-MAPK signaling following ARID1A loss were not addressed in this study. Future studies utilizing MAPK pathway inhibitors or conducting rescue experiments will be instrumental in comprehensively validating this functional requirement. Additionally, although small-molecule inhibitors targeting the RAS-MAPK pathway were not addressed in this study, comprehending the therapeutic potential of these inhibitors and clarifying the specific downstream signaling events subsequent to ARID1A loss are of crucial significance for advancing GISTs treatment. A limitation of the present study is its reliance on siRNA-mediated knockdown. Future investigations employing CRISPR/Cas9-mediated gene knockout and genetic rescue approaches in GIST models are currently being conducted in our laboratory and are anticipated to provide more definitive evidence.

## 5. Conclusions

In summary, ARID1A was decreased in GIST tissues with high-risk grade, and ARID1A suppresses GIST proliferation and metastasis by downregulating the expression of MEMO1 and inactivating ERK1/2 signaling pathway. Our findings illustrate that ARID1A might be a prognostic biomarker in GISTs and the MAPK signaling might be an effective therapeutic target for GIST patients.

## Supplementary Material

Supplementary figures and tables.

Supplementary table s3.

Supplementary table s4.

Supplementary table s5.

## Figures and Tables

**Table 1:** Association of expression levels of ARID1A with clinical features.

Characteristic	All patients	N%	ARID1A expression	ARID1A expression	*P*
			High (n = 58)	Low (n = 11)	
**Age (range), y**					
≤ 60	47	68.12	40	7	
> 60	22	31.88	18	4	0.734
**Gender**					
Male	40	57.97	36	4	
Female	29	42.03	22	7	0.182
**Tumor Location**					
gastric	32	46.38	30	2	
non-gastric	37	53.62	28	9	0.040*
**Tumor size(cm)**					
≤ 5	34	49.28	32	2	
> 5, ≤ 10	29	42.02	23	6	
> 10	6	8.70	3	3	0.020*
**Mitotic index**					
≤ 5/50hpf	50	72.46	43	7	
> 5/50hpf	19	27.54	15	4	0.478

## Abbreviations

GISTs: gastrointestinal stromal tumors
ICC: interstitial cells of Cajal
TKIs: tyrosine kinase inhibitors
SWI/SNF: switch/sucrose non-fermenting
AhR: aryl hydrocarbon receptor
TMA: tissue microarrays
TSS: transcription start site
qRT-PCR: quantitative reverse transcriptase-polymerase chain reaction
PCA: principal component analysis
KEGG: Kyoto encyclopedia of genes and genomes
DO: disease ontology
IHC: immunohistochemistry
