# Supplementary material for: Chromatin Remodeling Subunit ARID1A Negatively Regulates the Malignant Progression of Gastrointestinal Stromal Tumors by Targeting the MEMO1 Promoter
Source: Int J Med Sci. 2026 Aug 11;23(9):3027–43. doi: 10.7150/ijms.136256 (PMC13539913; doi:10.7150/ijms.136256)
Supplement: Supplementary file 1 [file ijmsv23p3027s1.pdf]

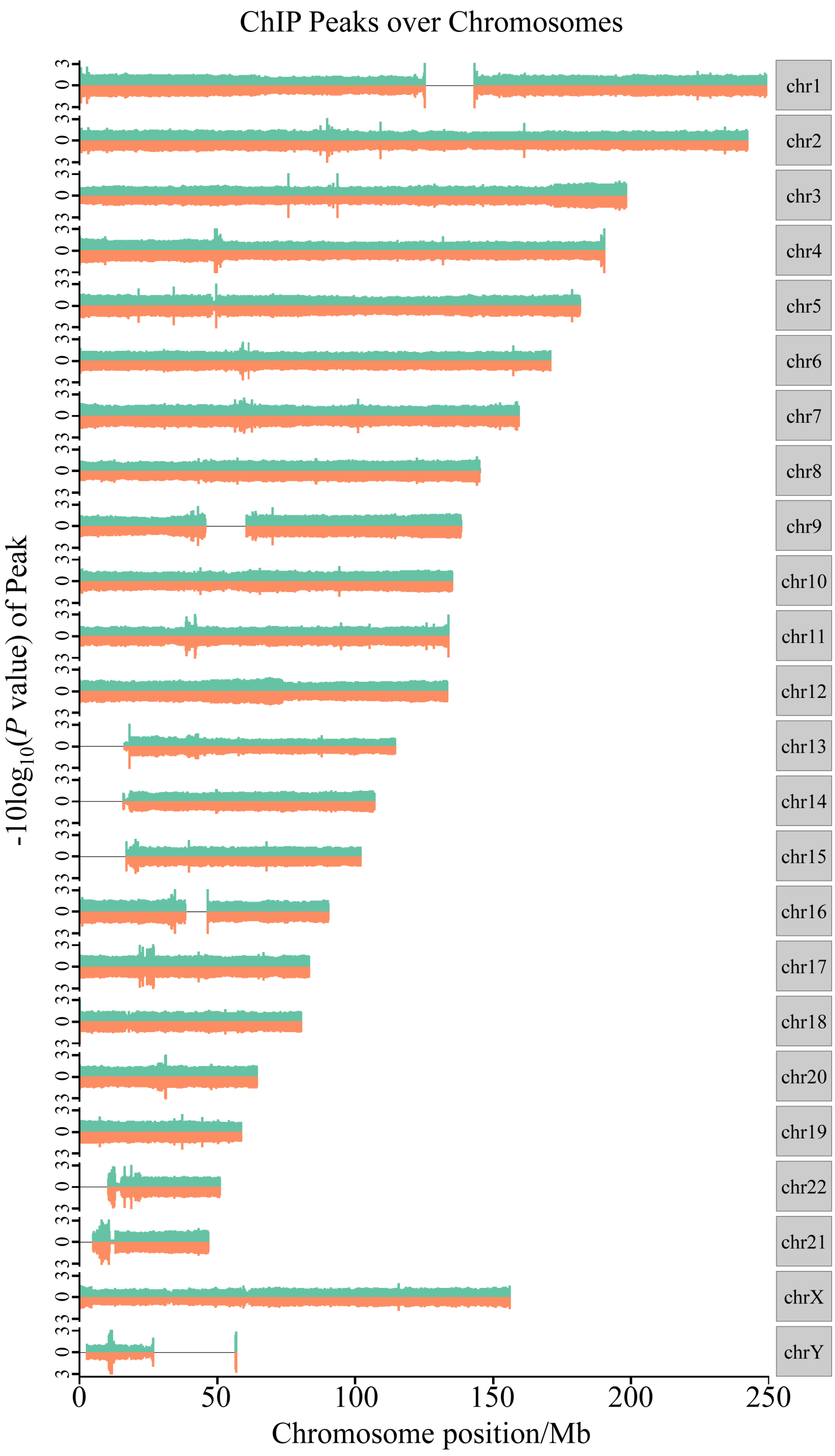

Supplementary Figure 1. Chromosome distribution of ARID1A ChIP-seq samples.

Figure. 1A

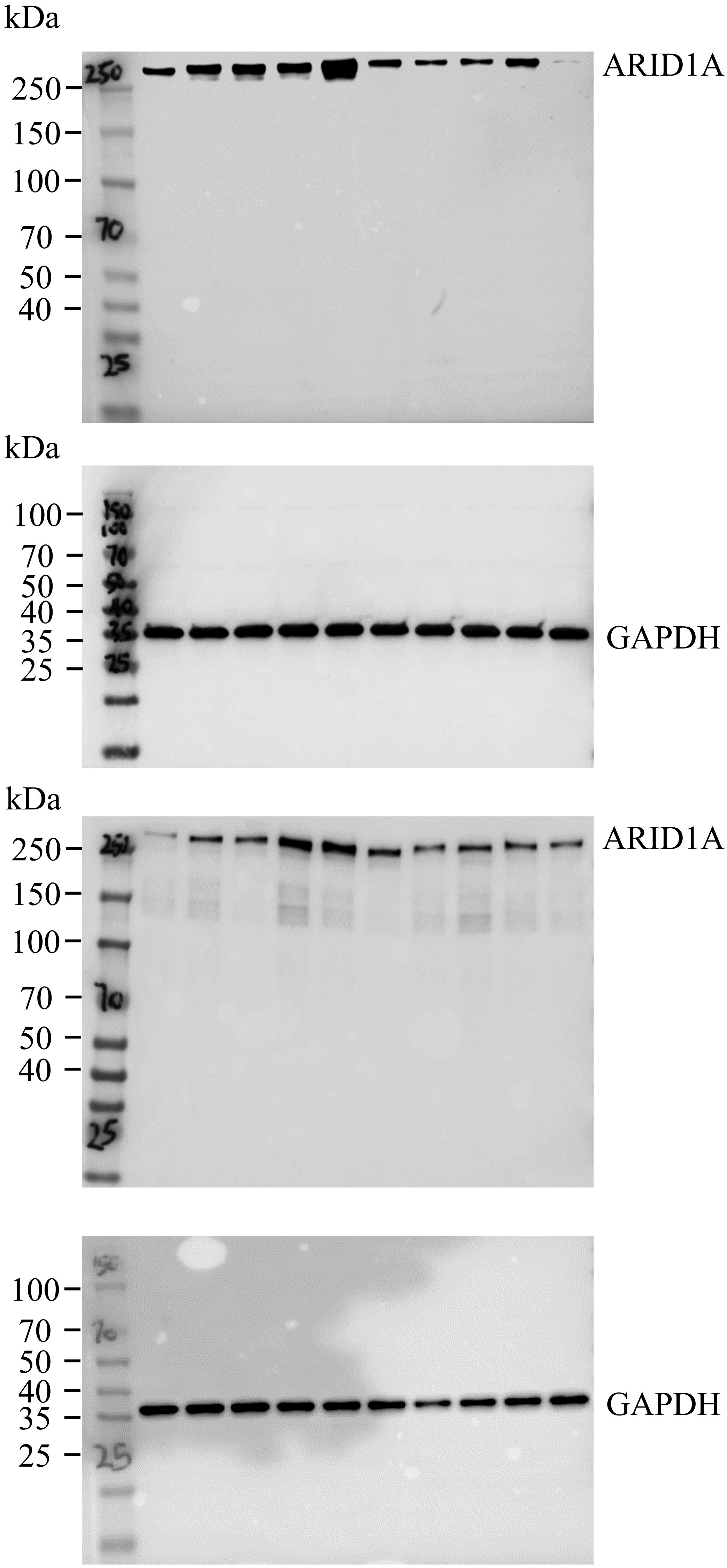

Supplementary Figure 2. The full uncropped Gels and Blots images

Figure. 2B

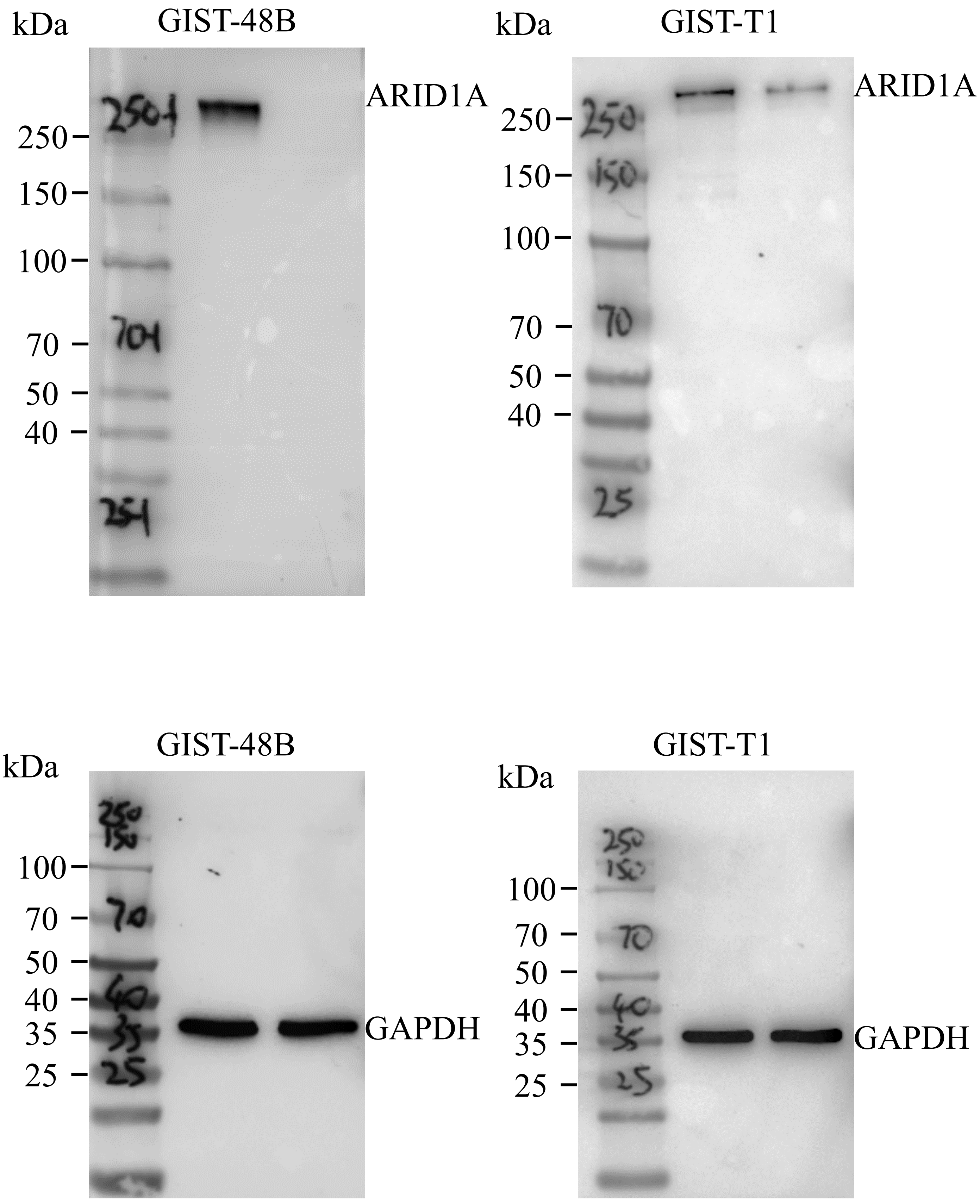

Supplementary Figure 2. The full uncropped Gels and Blots images

Figure. 3E

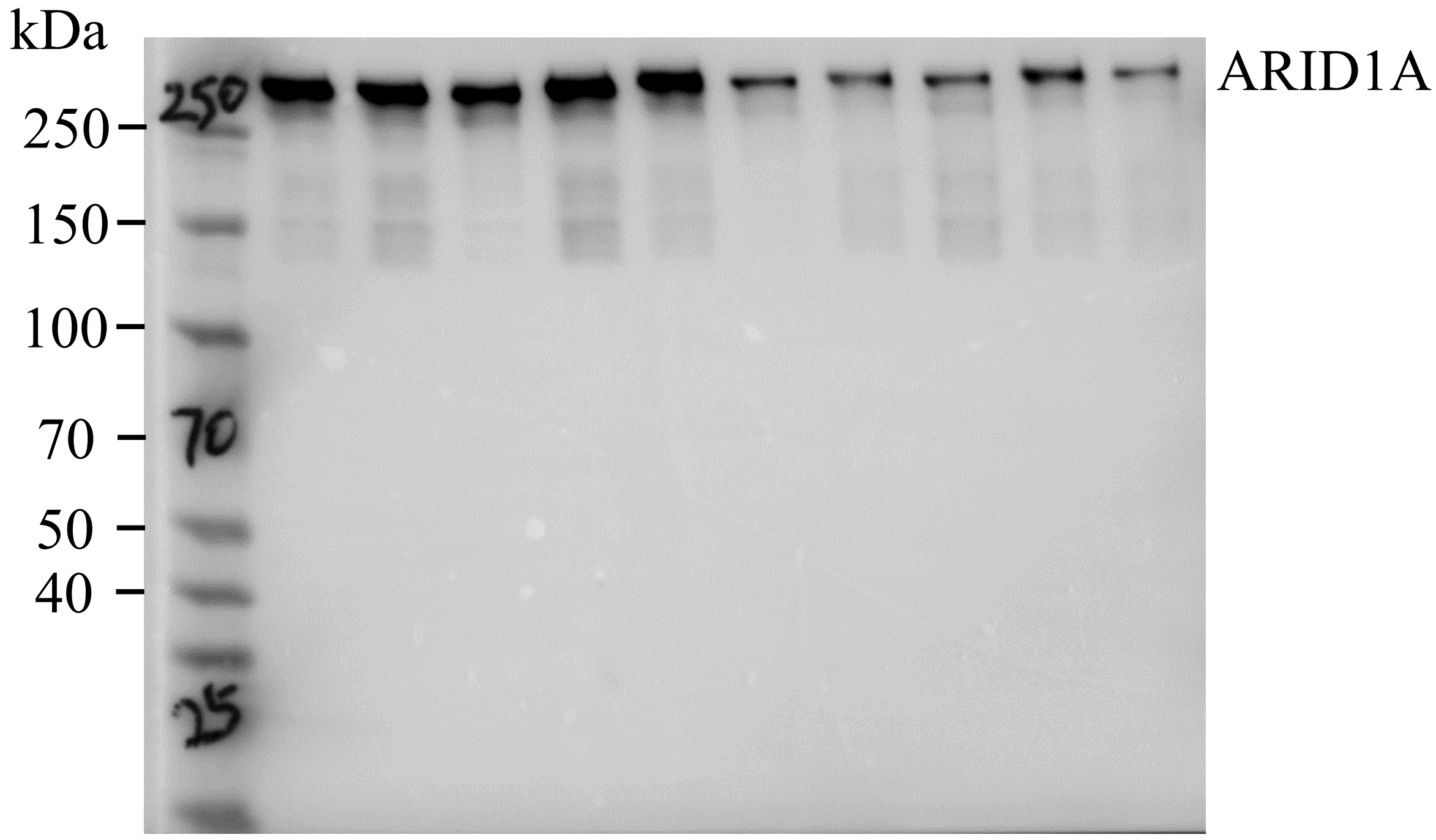

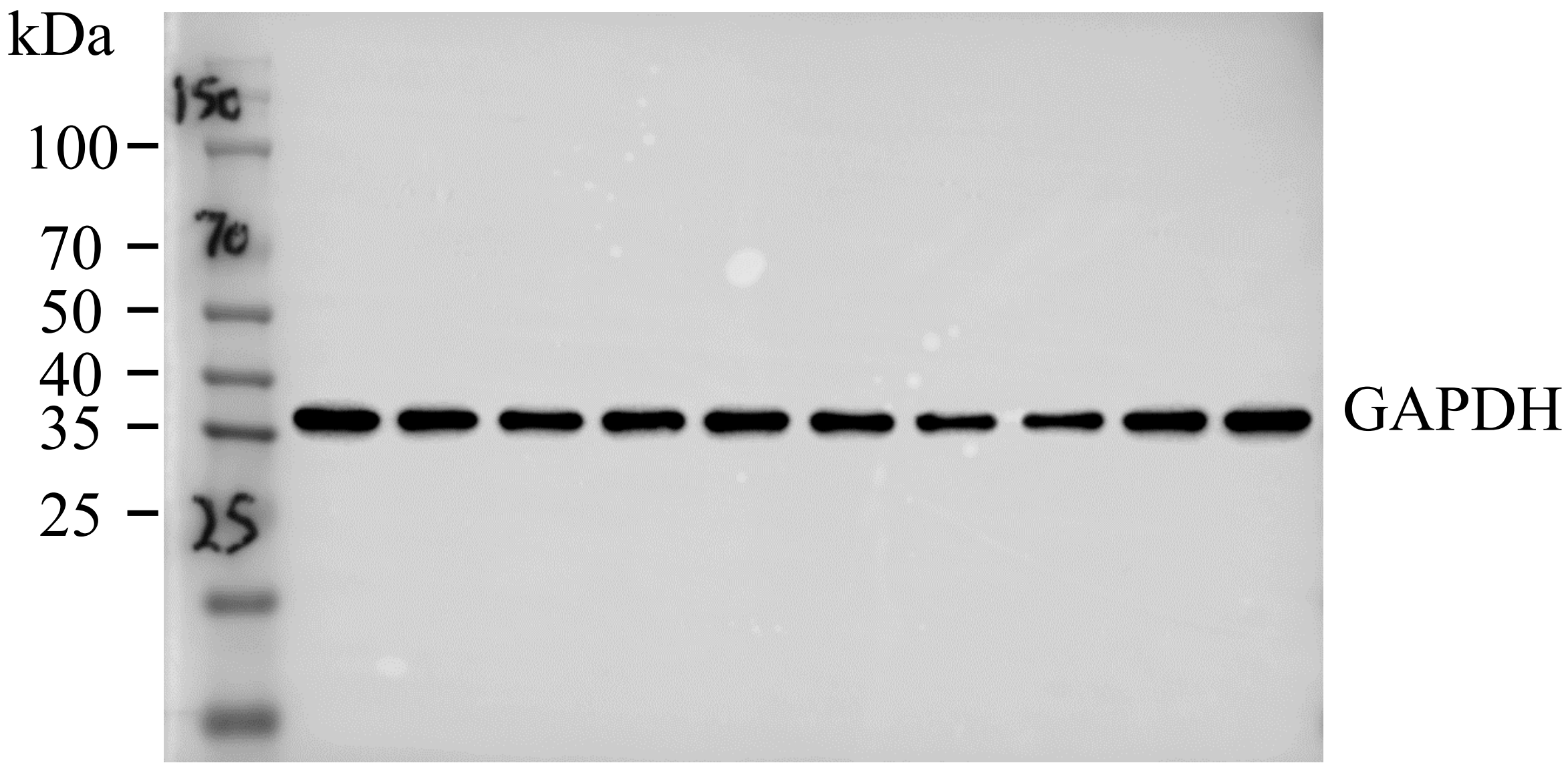

Supplementary Figure 2. The full uncropped Gels and Blots images

Figure. 5E

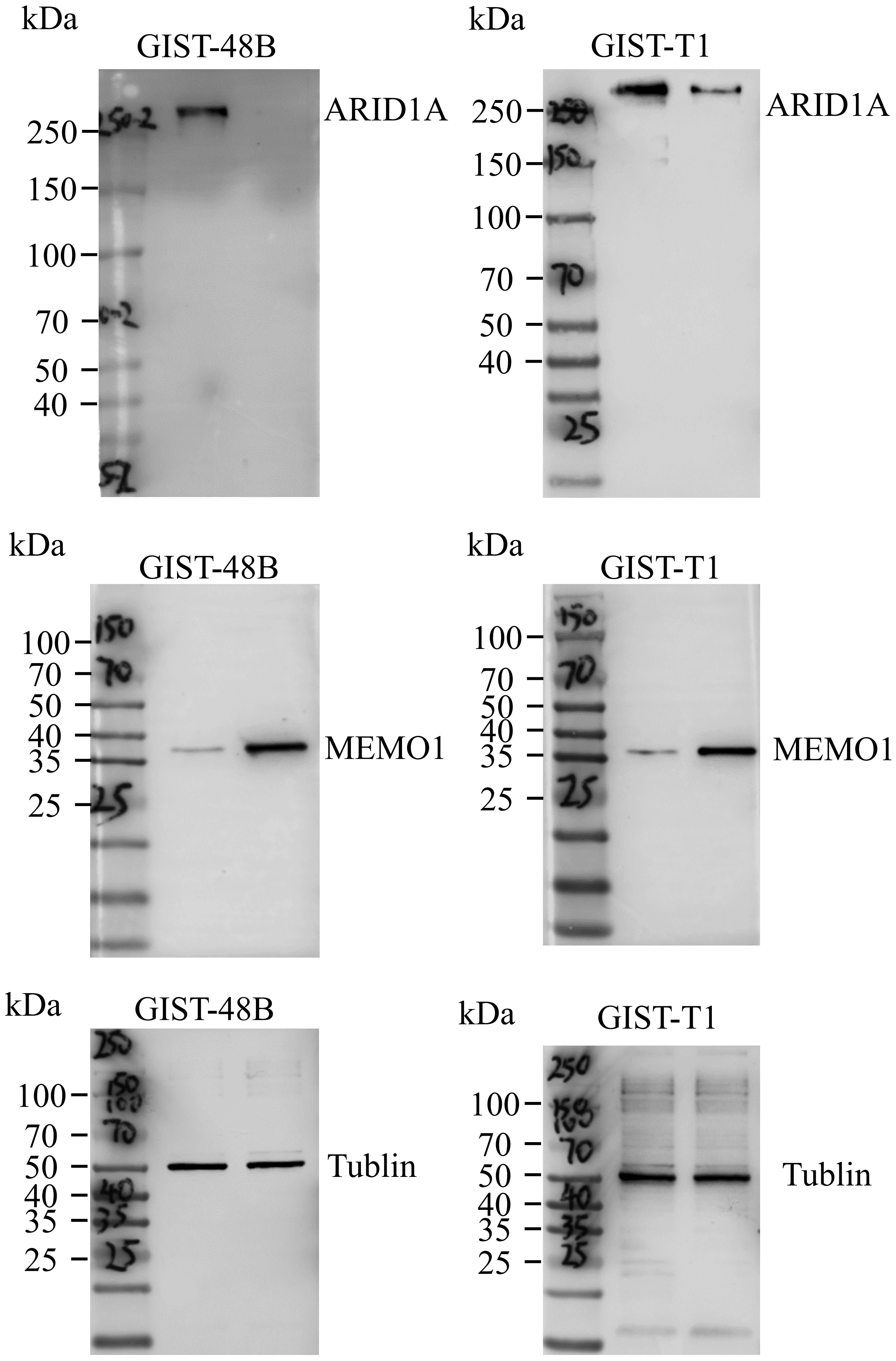

Supplementary Figure 2. The full uncropped Gels and Blots images

Figure. 6B

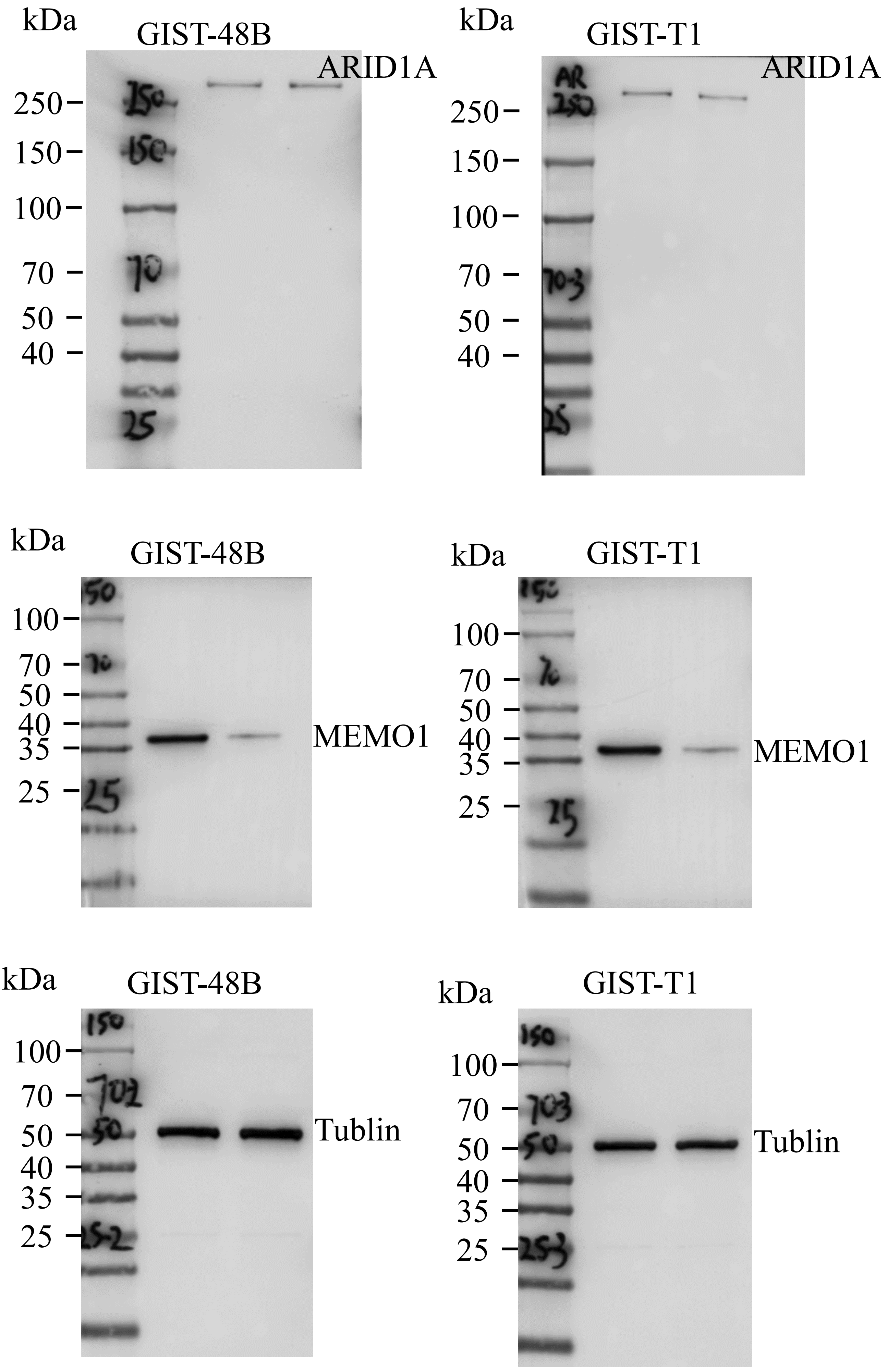

Supplementary Figure 2. The full uncropped Gels and Blots images

Figure. 7B

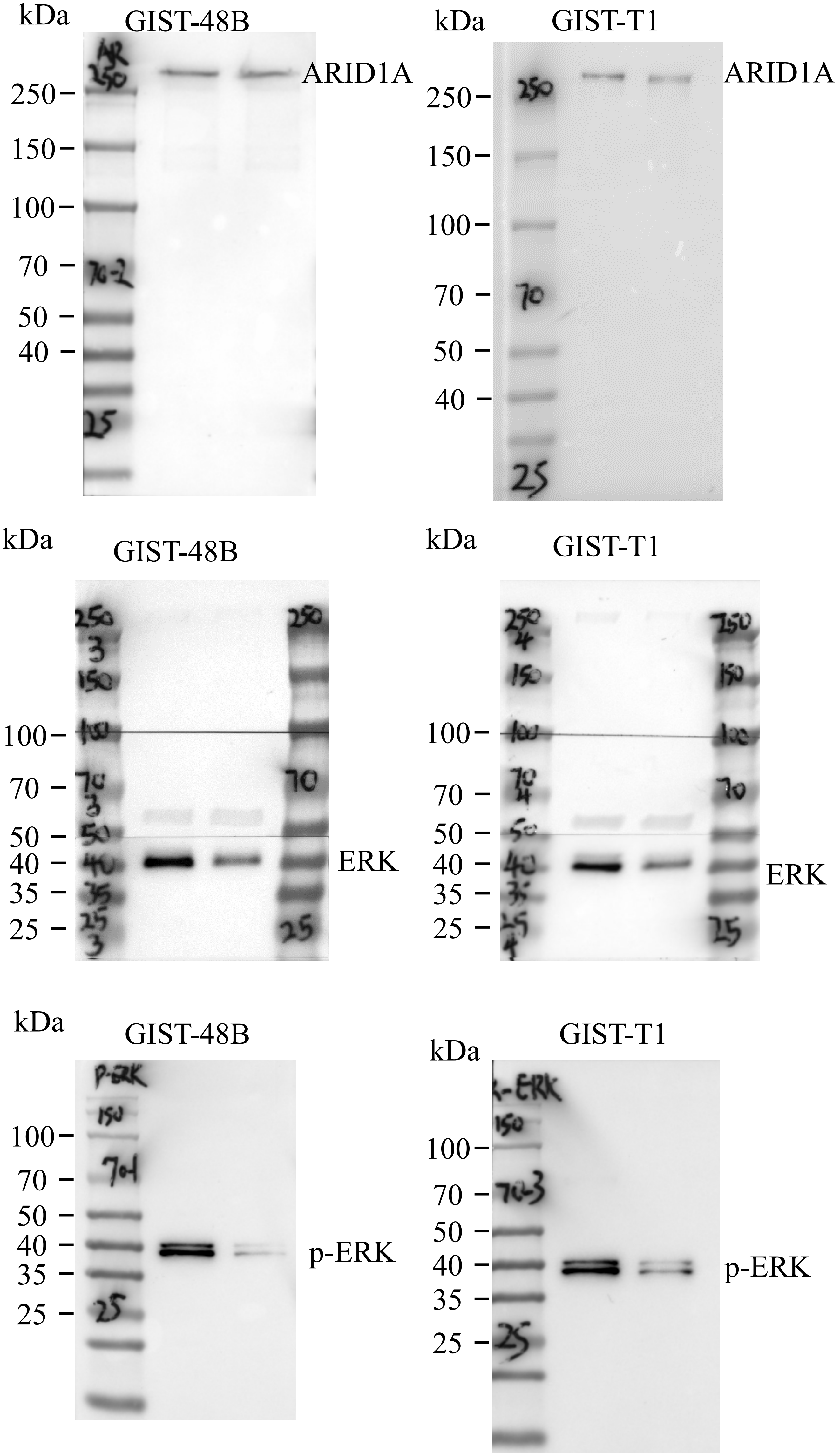

Supplementary Figure 2. The full uncropped Gels and Blots images

Figure. 7B

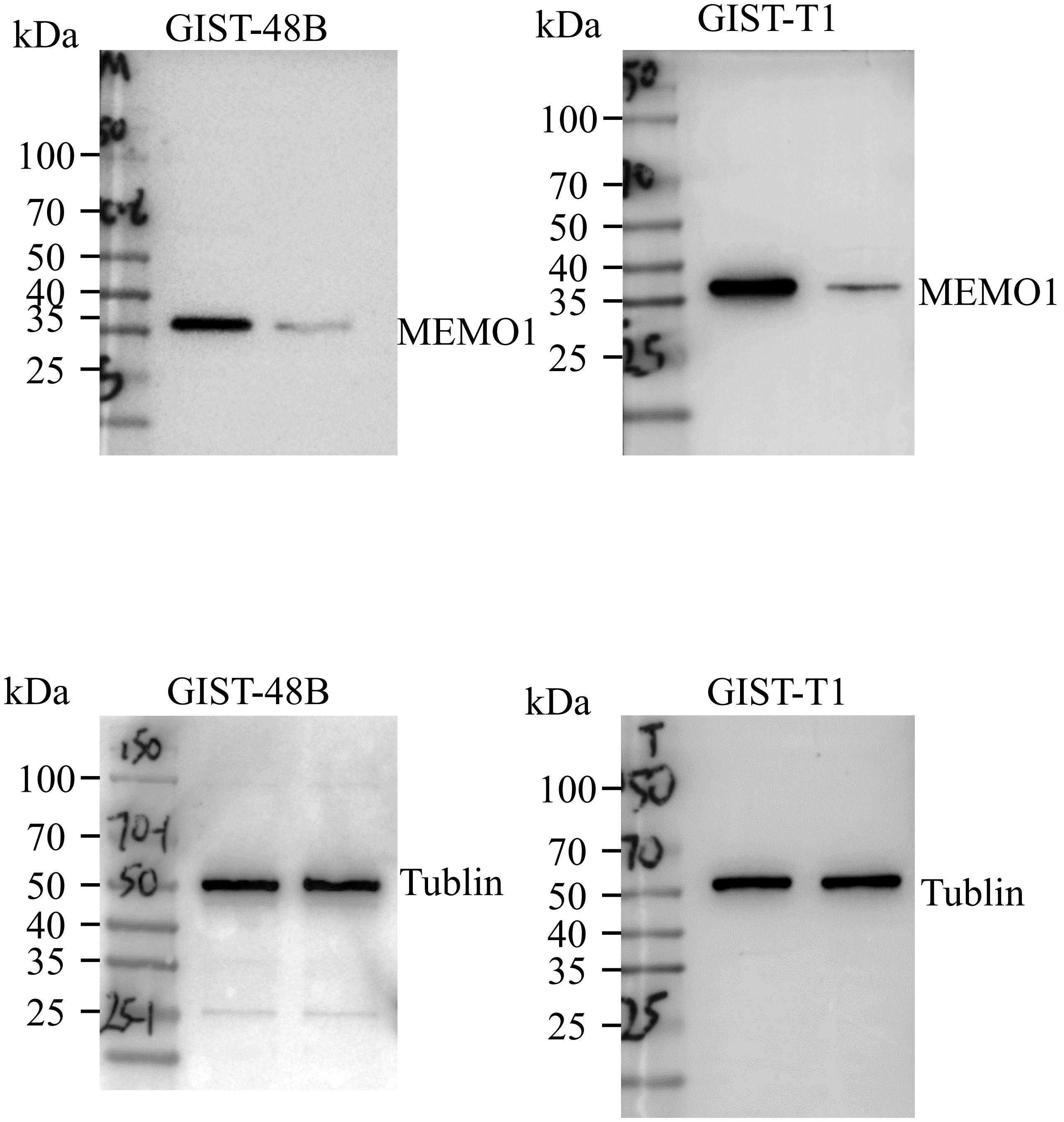

Supplementary Figure 2. The full uncropped Gels and Blots images

Supplementary Table 1. Multivariate Cox Regression Analysis of Clinicopathological Factors for OverallSurvival in GISTs Patients.

Characteristic	All patients(n=69)	N%	HR	95%CI	*P*
**Age (range), y**					
>60	22	31.88	1 [Reference]		
≤60	47	68.12	0.96	0.47-1.98	0.91
**Gender**					
Female	29	42.03	1 [Reference]		
Male	40	57.97	1.51	0.75-3.04	0.24
**Tumor Location**					
non-gastric	37	53.62	1 [Reference]		
gastric	32	46.38	0.64	0.30-1.36	0.24
**Tumor size(cm)**					
>10	6	8.70	1 [Reference]		
>5，≤10	29	42.02	0.57	0.21-1.56	0.27
≤5	34	49.28	0.15	0.04-0.58	0.01
**Mitotic index**					
>5/50hpf	19	27.54	1 [Reference]		
≤5/50hpf	50	72.46	0.37	0.15-0.91	0.03
**ARID1A status**					
low expression	11	15.94	1 [Reference]		
high expression	58	84.06	0.34	0.14-0.81	0.01

HR, hazard ratio; CI, confidence interval.

Supplementary Table 2. Sequence of primers used in this study.

Primers for qRT-PCR assy.

Gene	Primer sequences (5' to 3')
ACTIN	Forward: CATGTACGTTGCTATCCAGGC
	Reverse: CTCCTTAATGTCACGCACGAT
GAPDH	Forward: AGAAGGCTGGGGCTCATTTG
	Reverse: AGGGGCCATCCACAGTCTTC
ARID1A	Forward: AACATGGCGGACAACAAAGC
	Reverse: TGGTCGTGAGAGTTCTGCTG
MEMO1	Forward: TTGGGCCTTCTCATCATGTGC
	Reverse: TGCGTTCAAACATTCCTGTCT

Primers for siRNA sequences.

Gene	Primer sequences (5' to 3')
ARID1A	Forward: GCAGGAGCUAUCUCAAGAUTT
	Reverse: AUCUUGAGAUAGCUCCUGCTT
MEMO1	Forward: GCGGAUCCUAGUAAUCUCUTT
	Reverse: AGAGAUUACUAGGAUCCGCTT
Negative control	Forward: UUCUCCGAACGUGUCACGUTT
	Reverse: ACGUGACACGUUCGGAGAATT

Primers for PCR after ChIP assay.

	Primer sequences (5' to 3')
MEMO1 promotor	Forward: TAGGACCACAGGCGAAGC
-1000~-750bp site 1#	Reverse: GCCTATTCCCAGCACTTT
MEMO1 promotor	Forward: AGAGGTCCAAGATAGCCTCCT
-750~-500bp site 2#	Reverse: GTGAACAGCCTGACAGGGTAA
MEMO1 promotor	Forward: GGTGGTAAGAGTGACCCTGT
-500~-250bp site 3#	Reverse: CTCATTCCTAGGGTGGCACG
MEMO1 promotor	Forward: CGTGCCACCCTAGGAATGAG
-250~0bp site 4#	Reverse: TCCCGCGGGTATCCATCATA
MEMO1 bind site	Forward: TTGAACCTCCCTCAAGAGCA
site 5#	Reverse: CACCACGCCAGCTAATTTTT
MEMO1 bind site	Forward: ACGAGGTCAGGAGATCGAGA
site 6#	Reverse: AGTGCAGTGGCACAATCTCA
MEMO1 bind site	Forward: GTGTTCGAGACCAGCCTAGC
site 7#	Reverse: ATCTTGGCTCACTGCAACCT
